# What makes a reaction network “chemical”?

**DOI:** 10.1186/s13321-022-00621-8

**Published:** 2022-09-19

**Authors:** Stefan Müller, Christoph Flamm, Peter F. Stadler

**Affiliations:** 1 Faculty of Mathematics, University of Vienna, Oskar-Morgenstern-Platz 1, 1090 Vienna, Austria; 2 Department of Theoretical Chemistry, University of Vienna, Währinger Straße 17, 1090 Vienna, Austria; 3 Bioinformatics Group, Department of Computer Science, and Interdisciplinary Center for Bioinformatics, Universität Leipzig, Härtelstraße 16–18, 04107 Leipzig, Germany; 4 German Centre for Integrative Biodiversity Research (iDiv) Halle-Jena-Leipzig & Competence Center for Scalable Data Services and Solutions Dresden-Leipzig & Leipzig Research Center for Civilization Diseases University Leipzig, 04107 Leipzig, Germany; 5 Max Planck Institute for Mathematics in the Sciences, Inselstraße 22, 04103 Leipzig, Germany; 6 Faculdad de Ciencias, Universidad Nacional de Colombia, Sede Bogotá, Ciudad Universitaria, Bogotá, 111321 Colombia; 7 Santa Fe Institute, 1399 Hyde Park Rd., Santa Fe, NM87501 USA

**Keywords:** Chemical reaction network, Directed hypergraph, Stoichiometric matrix, Futile cycle, Perpetuum mobile, Energy conservation, Mass conservation, Reaction invariants, Null spaces, Sum formula, Multigraph, Lewis formula

## Abstract

**Background:**

Reaction networks (RNs) comprise a set *X* of species and a set $\mathscr {R}$ of reactions $Y\rightarrow Y'$, each converting a multiset of educts $Y\subseteq X$ into a multiset $Y'\subseteq X$ of products. RNs are equivalent to directed hypergraphs. However, not all RNs necessarily admit a chemical interpretation. Instead, they might contradict fundamental principles of physics such as the conservation of energy and mass or the reversibility of chemical reactions. The consequences of these necessary conditions for the stoichiometric matrix $\mathbf {S}\in \mathbb {R}^{X\times \mathscr {R}}$ have been discussed extensively in the chemical literature. Here, we provide sufficient conditions for $\mathbf {S}$ that guarantee the interpretation of RNs in terms of balanced sum formulas and structural formulas, respectively.

**Results:**

Chemically plausible RNs allow neither a perpetuum mobile, i.e., a “futile cycle” of reactions with non-vanishing energy production, nor the creation or annihilation of mass. Such RNs are said to be thermodynamically sound and conservative. For finite RNs, both conditions can be expressed equivalently as properties of the stoichiometric matrix $\mathbf {S}$. The first condition is vacuous for reversible networks, but it excludes irreversible futile cycles and—in a stricter sense—futile cycles that even contain an irreversible reaction. The second condition is equivalent to the existence of a strictly positive reaction invariant. It is also sufficient for the existence of a realization in terms of sum formulas, obeying conservation of “atoms”. In particular, these realizations can be chosen such that any two species have distinct sum formulas, unless $\mathbf {S}$ implies that they are “obligatory isomers”. In terms of structural formulas, every compound is a labeled multigraph, in essence a Lewis formula, and reactions comprise only a rearrangement of bonds such that the total bond order is preserved. In particular, for every conservative RN, there exists a Lewis realization, in which any two compounds are realized by pairwisely distinct multigraphs. Finally, we show that, in general, there are infinitely many realizations for a given conservative RN.

**Conclusions:**

“Chemical” RNs are directed hypergraphs with a stoichiometric matrix $\mathbf {S}$ whose left kernel contains a strictly positive vector and whose right kernel does not contain a futile cycle involving an irreversible reaction. This simple characterization also provides a concise specification of random models for chemical RNs that additionally constrain $\mathbf {S}$ by rank, sparsity, or distribution of the non-zero entries. Furthermore, it suggests several interesting avenues for future research, in particular, concerning alternative representations of reaction networks and infinite chemical universes.

**Supplementary Information:**

The online version contains supplementary material available at 10.1186/s13321-022-00621-8.

## Background

Most authors will agree that a chemical reaction network consists of a set *X* of chemical species or compounds and a set $\mathscr {R}$ of chemical reactions, each describing the transformation of some (multi)set of educts into a (multi)set of products. Depending on the application, this basic construction may be augmented by assigning properties such as mass, energy, sum formulas, or structural formulas to the compounds. Similarly, reactions may be associated with rate constants, equilibrium constants, and so on. A formal theory of reaction networks (RN) describes a reaction on a given set of compounds *X* as a *stoichiometric relation*, i.e., as a pair of formal sums of chemical species $x \in X$:

1 $$\begin{aligned} \sum _{x\in X} s^-_{xr} \, x \rightarrow \sum _{x\in X} s^+_{xr} \, x . \end{aligned}$$

The left-hand side in Eq. (1) lists the educts and the right-hand side gives the products of the reaction. The *stoichiometric coefficients* $s^-_{xr}\in \mathbb {N}_0$ and $s^+_{xr}\in \mathbb {N}_0$ denote the number of species $x\in X$ that are consumed (on the left-hand side) or produced (on the right-hand side) by the reaction *r*, respectively. A species $x\in X$ is an educt in reaction *r* if $s^-_{xr}>0$ and a product if $s^+_{xr}>0$. If $s^+_{xr}=s^-_{xr}=0$, then species *x* does not take part in reaction *r* and is suppressed in the conventional chemical notation. The formal sums $\sum _{x\in X} s^-_{xr} \, x$ and $\sum _{x\in X} s^+_{xr} \, x$ form the *complexes* of educts $r^-$ and products $r^+$ of the reaction *r*. We denote the set of reactions under considerations by $\mathscr {R}$ and call the pair $(X,\mathscr {R})$ a reaction network (RN). Throughout this contribution we will assume that both *X* and $\mathscr {R}$ are non-empty and finite. Excluding explicit catalysis, that is, forbidding $s^-_{xr} \, s^+_{xr}>0$, it suffices to consider the *stoichiometric matrix* $\mathbf {S}\in \mathbb {N}_0^{X \times \mathscr {R}}$. Its entries $\mathbf {S}_{xr} = s^+_{xr} - s^-_{xr}$ describe the net production or consumption of species *x* in reaction *r*. In many practical applications, e.g. in the context of metabolic networks, RNs are embedded in an open system. In that manner, the consumption of nutrients and the production of waste can be modeled. We will return to this point only after discussing chemical RNs in isolation, i.e., as closed systems.

Several graph representations have been considered as (simplified) models of a RN, see [1] for a recent summary. In contrast to the pair $(X,\mathscr {R})$, they do not always completely represent the RN.

The S-graph (*species graph*, *compound graph*, or *substrate network* in the context of metabolic networks) has the species as its vertices. A (directed) edge connects *x* to *y* if the RN contains a reaction that has *x* as an educt and *y* as a product [2, 3]. The corresponding construction in the kinetic setting is the *interaction* graph with undirected edges whenever $\partial [x]/\partial [y]\not \equiv 0$, which are usually annotated by the sign of the derivative [4]. S-graphs have also proved to be useful in approximation algorithms for the minimal seed set problem [5], which asks for the smallest set of substrates that can generate all metabolites. Complementarily, *reaction graphs* model reactions as nodes, while edges denote shared molecules [6].

The *complex-reaction graph* simply has the complexes $\mathscr {C}$ (the left- and right-hand sides of the reactions) as its vertex set and the reactions $\mathscr {R}$ as its edge set. That is, two complexes $r^-$ and $r^+$ are connected by a directed edge if there is a reaction $r=(r^-,r^+)\in \mathscr {R}$. Its incidence matrix $\mathbf {Z} \in \mathscr {R}^{\mathscr {C}\times \mathscr {R}}$ (with entries $\mathbf {Z}_{cr}=-1$ if $c=r^-$, $\mathbf {Z}_{cr}=1$ if $c=r^+$, and $\mathbf {Z}_{cr}=0$ otherwise) is linked to the stochiometric matrix via $\mathbf {S}=\mathbf {Y}\mathbf {Z}$, where the entries of the (stoichiometric) complex matrix $\mathbf {Y} \in \mathbb {R}^{X \times \mathscr {C}}$ are the corresponding stochiometric coefficients. The complex-reaction graph plays a key role in the analysis of chemical reaction networks with mass-action kinetics and arbitrary rate constants, as studied in classical “chemical reaction network theory” (CRNT) [7–9]. It gives rise to notions such as “complex balancing” and “deficiency”, which allow the formulation of strong (global) stability results, see e.g. [10, 11].

SR-graphs (*Species-reaction networks*) are bipartite graphs with different types of nodes for chemical species and reactions, respectively [12, 13]. As such, they can be endowed with additional annotations or extended with multiple edges to represent stoichiometric coefficients. In this extended form, they are faithful representations of chemical RNs. Alternatively, the edges are often annotated with the multiplicities of molecules, i.e., the stoichiometric coefficients; in this case, they completely specify the RN $(X,\mathscr {R})$. Undirected SR-graphs have a close relationship to classical deficiency theory [7, 9] and form the starting point for a qualitative theory of chemical RN kinetics [14]). More detailed information on qualitative kinetic behavior can be extracted from directed SR-graphs [15]. Both the S- and the R-graph can be extracted unambigously from an SR-graph.

The bipartite SR-graphs can be interpreted as the König’s representation [16] of directed hypergraphs. The connection between hypergraph and graph representations is discussed in some more detail in [17]. While SR-graphs and directed hypergraphs can be transformed into each other, they carry a very different semantic. For instance, the notions of path and connectivity are very different for bipartite graphs and directed hypergraphs [18]. It has been argued, therefore that any graph representation of chemical networks necessarily treats edges as independent entities and thus fails to correctly capture the nature of chemical reactions [19, 20]. In a similar vein, [21] adopts the hypergraph representation and models (bio)chemical pathways as integer hyperflows to ensure mass balance at each vertex. Not every pair of an S- and R-graph implies an SR-graph, and if they do, the result need not be unique [6].

Over the last decade, many authors, including one of us, have investigated metabolic networks from a statistical perspective and reached the conclusion that they are distictly “non-random”, presumably as the consequence of four billion years of evolution. This conclusion is typically reached by first converting a RN into one of the graph representations mentioned above. The choice of graphs is largely motivated by a desire to place metabolic or other chemical RNs within the scheme of small world and scale free networks and to analyze the RNs with the well-established tools of network science [19, 22]. Thus one concludes that graph-theoretical properties of metabolic networks are significantly different from the properties of randomly generated or randomized background models for chemical reaction networks [3, 23–25]. The insights gained from this “non-randomness” of metabolism, however, critically depend on what exactly the authors meant by “random”, that is, how the background models are defined. In particular, it is important to understand whether differences between chemical networks and the background are caused by the implementation of universal properties (that any “chemistry-like” RN must satisfy) or whether they arise from the intrinsic structure of particular chemical networks.

To this end, however, we first need a comprehensive conception of what constitutes a *chemistry-like* reaction network. The different representations used in the literature highlight the fact that it is far from obvious which graphs or hypergraphs properly describe *chemical* RNs among a possibly much larger set of network models. There is a significant body of work in the literature that describes necessary conditions on the stoichiometric matrix $\mathbf {S}$ that derive from key properties of chemical RNs, such as the conservation of mass or atoms in each reaction [8, 26–30]. In contrast, we are interested here in sufficient conditions with the aim of providing a concise characterization of RNs $(X,\mathscr {R})$ and their stoichiometric matrices $\mathbf {S}$ that describe reaction system that can reasonably be considered as “chemistry-like”. This is of practical relevance in particular for the construction of artifical chemistry models [31–34] and random “chemistries”: It is still an open problem how random RNs can be constructed that can serve as fair, chemistry-like background models. We therefore start with a brief survey of random artificial chemistries and randomized RNs. As we shall see in the following section, oftentimes no explicit provisions are made to include “chemical” constraints such as the conservation of matter and energy into the background models.

Beyond the practical importance for the generation of random chemistries, it is also of interest to ask whether and to what extent the stoichiometry of a RN constrains the underlying chemistry, i.e., the composition of compounds and the type of reactions. Chemical reaction networks have been studied as a paradigm of computation that is quite different from, but theoretically equally powerful as Turing machines [35–38]. In the case of DNA based computing [39], the field has matured to the point that a compiler for translating chemical reaction networks into nucleic acid strand displacement systems has become available [40]. If chemical reaction networks are to be used as computing devices, a necessary intermediate step is to design reaction systems that implement a given stoichiometric matrix. Constraints on the chemistry imposed by the desired network stoichiometry itself thus become an issue in the design process, prompting us to ask whether there are chemical limitations to the realizability of RNs also beyond the constraints imposed by thermodynamics.

The main part of this contribution is the characterization of chemistry-like RNs. Starting from the principles of energy conservation and conservation of matter, we derive equivalent conditions on the stoichiometric matrix $\mathbf {S}$. We then introduce realizability of RNs by sum formulas and structural formulas as a first step towards a formalization of chemistry-like networks, and show that conservation of matter is already sufficient to guarantee the existence of such chemistry-like representations. Finally we discuss the consequences of the mathematical results for the construction of random RNs and address some open research questions.

## A brief survey of random and randomized chemical RNs

Chemical reaction networks are specified either as a set of chemical reactions or as a system of differential equations describing its kinetics. Graphical models have been extracted from both.

### Simple graph models of RNs

S-graphs have been used to explore statistical properties of large RNs. In this line of research, empirical S-graphs are compared to the “usual” random networks models such as Erdős Renyí (ER) random graphs, Small World networks in the sense of Watts and Strogatz [41], or the Álbert-Barabasi model of preferential attachment. Generative models for random graphs with given degree distributions were introduced in [42]. Not surprisingly, chemical reaction networks do not very well conform to either one of them. As noted early on, however, R-graphs of metabolic networks at least qualitatively fit the small world paradigm [22]. More sophisticated analyses detected evidence for modularity and hierarchical organization in metabolic networks [43], using random graph models with the same degree distributions as contrasts. Arita noted, however, that S-graphs are poor representations of biochemical pathways and proposed an analysis in terms of atom traces, concluding that “the metabolic world [of *E. coli*] is not small in terms of biosynthesis and degradation” [44]. The motivation to focus on atom maps comes from the insight that two compounds that are linked by reactions are only related by the chemical transformation if they share at least one atom.

A versatile generator for bipartite graphs that can handle joint degree distributions is described in [45]. Surprisingly, bipartite random graph models apparently have not been used to model chemistry. Instead of generative models such as the ER graph or the preferential attachment model, null models are often specified in terms of rewiring, that is, edit operations on the graph. Rewiring rules define a Markov Process on a set of graphs that can produce samples of randomized networks. The key idea is to specify the rewiring procedure in such a way that it preserves graph properties that are perceived to be important [46, 47]. For example, the degrees of all vertices in a digraph are preserved when a pair of directed edges $x_1y_1$ and $x_2y_2$ is replaced by $x_1y_2$ and $x_2y_1$ as long as $x_1$ and $x_2$ have the same out-degree while $y_1$ and $y_2$ have the same in-degree. Randomization procedures for bipartite graphs have become available in the context of ecological networks [48] or trade networks [49]. To our knowledge they have not been used for SR graphs.

### Random (directed) hypergraphs

In [50] a hypergraph is defined as a multiset of hyperedges, each of which in turn is a multiset of vertices. In this setting, a random hypergraph is specified by the probabilities $p_k$ to include a hyperedge *e* with cardinality $|e|=k$. Similar models for undirected hypergraphs are used e.g. in [51]. In a directed hypergraph, every hyperedge is defined as the pair $(e_-,e_+)$ consisting of the multisets $e_-$ and $e_+$. The construction of [50] thus naturally generalizes to directed hypergraphs specified by picking *e* with probability $p_{|e_-|,|e_+|}$. In the context of chemistry this amounts to picking educt and product sets for reactions with probabilities depending on their cardinality. This type of random (directed) hypergraph models are the obvious generalizations of the Erdős Renyí (di)graphs. A certain class of random directed hypergraphs with $|e^-|=2$ and $|e^+|=1$ for all hyperedges *e* is considered in [52].

Hypergraphs are also amenable to rewiring procedures that ensures the preservation of certain local or global properties. For instance [17] proposes a scheme that preserves the number and cardinality of the hyperedges (replacing a randomly selected $(e_-,e_+)$ with a randomly selected pair of disjoint subsets $(e_-',e_+')$ with $|e_-|=|e_-'|$ and $|e_+|=|e_+'|$). On this basis, the authors conclude that the hierarchical structure hypothesis proposed in [43] is not supported for metabolic networks when a clustering coefficient is defined for directed hypergraphs. [17] also compares S- and R-graphs of metabolic networks with ensembles of S- and R-graphs derived from randomized directed hypergraphs and cast further doubt on previously reported scaling results. Randomization procedures for hypergraphs that preserve local clustering are described in [53]. An approach that uses a chemical graph rewriting model to ensure soundness of reactions is described in the MSc thesis [54].

In [25] networks are constructed in a stepwise procedure starting with directed graphs whose arcs are then re-interpreted as directed hyperarcs by combining multiple arcs. This process is guided by matching the degree distribution of the implied S-graph.

### Reaction universes: random subhypergraphs

Instead of generating a random RN directly from a statistical model or rewiring a given one, one can also start from a *reaction universe* RU, that is, a RN that contains all species of interest and all known or inferred reactions between them. Without losing generality we can think of the RU as a directed hypergraph in the sense of [50], where the multi-set formalism accounts for the stoichiometric coefficients. In contrast to the generative and rewiring approaches the *a priori* specification of an RU ensures a high level of chemical realism and RNs can now be sampled by randomly selecting subsets of directed hyperedges, that is, chemical reactions. If the RU already ensures conservation of matter or energy, these properties are inherited by the sub-networks. In order to generate random metabolic networks, reactions can be drawn from databases such as KEGG or EcoCyc [55, 56]. Such selections of reactions are sometimes called “metabolic genotypes” since the available reactions are associated with enzymes, whose presence or absence is determined by an organism’s genome [55]. In some studies, additional constraints such as the production of biomass are exploited and networks are sampled e.g. by combining Flux-Balance Analysis (FBA) and a Markov Chain Monte Carlo (MCMC) approach [55, 57].

## A characterization of chemistry-like reaction networks

In this section, we start from reaction networks that are specified as abstract stoichiometric relations, Eq. (1), and identify minimal constraints necessary to avoid blatantly unphysical behavior.

### Notation and peliminaries

Let *X* be a finite set and let $\mathscr {R}$ be a pair of formal sums of elements of *X* with non-negative integer coefficients according to Eq. (1). Then we call the pair $(X,\mathscr{R})$ a *reaction network* (RN). Equivalently, a RN is a directed, integer-weighted hypergraph with directed edges $(r^-,r^+)$ such that $x\in r^-$ with weight $s^-_{xr}>0$ and $x\in r^+$ with weight $s^+_{xr}>0$. The weights $s^-_{xr}$ and $s^+_{xr}$ are usually called the *stoichiometric coefficients*. We set $s^-_{xr}=0$ and $s^+_{xr}=0$ if $x\notin r^-$ and $x\notin r^-$, respectively. We deliberately dropped the qualifier *chemical* here since, as we shall see, not every RN $(X,\mathscr {R})$ makes sense as a model of a chemical system. In fact, the aim of this contribution is to characterize the set of RNs that make sense as models of chemistry.

**Fig. 1:**
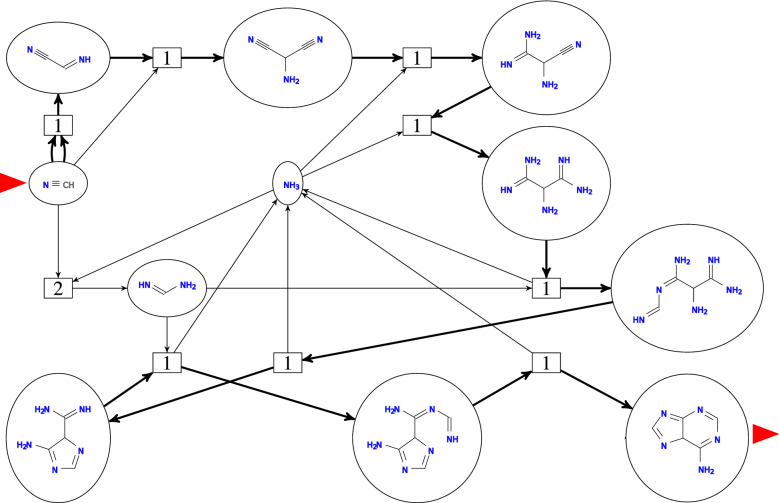
Representation of a RN as König multigraph of the corresponding directed hypergraph. Round vertices (with chemical structures shown inside) designate compounds $x\in X$, while reactions $r\in R$ are shown as square vertices. Stoichometric coefficients are indicated by the number of edges from *x* to *r* for $s^-_{xr}>0$ and *r* to *x* for $s^+_{xr}>0$, respectively. A flow (an overall reaction) is given by non-negative integer multiples of individual reactions. Here the coefficients $v_r$ are indicated in the square nodes for each reaction *r*. The flow shown here defines Oró’s [58] route from *HCN* to adenine (marked by red triangles) and corresponds to the net reaction ${5 HCN \longrightarrow H5C5N5}$. Figure adapted from [59]

Such directed hypergraphs are most conveniently drawn as (bipartite) König multigraphs, with distinct types of vertices representing compounds $x\in X$ and reactions $r\in \mathscr {R}$, respectively. Stoichiometric coefficients larger than one appear as multiple edges. See the example in Fig. 1.

For each reaction $r\in \mathscr {R}$, we define its support as ${{\,\mathrm{supp}\,}}(r)=\{x \mid s^-_{xr}+s^+_{xr}>0\}$; that is, $x\in {{\,\mathrm{supp}\,}}(r)$ if it appears as an educt, a product, or a catalyst in *r*. The stoichiometric matrix of $(X,\mathscr {R})$ is $\mathbf {S}\in \mathbb {N}_0^{X \times \mathscr {R}}$ with entries $\mathbf {S}_{xr}= s^+_{xr} - s^-_{xr}$.

We distinguish *proper reactions* *r*, for which there is both $x\in X$ with ${\mathbf {S}}_{xr}<0$ and $y\in X$ with ${\mathbf {S}}_{yr}>0$, *import reactions* for which ${\mathbf {S}}_{xr}\ge 0$ for all $x\in X$, and *export reactions* for which ${\mathbf {S}}_{xr}\le 0$ for all $x\in X$. We write $\varnothing$ for the empty formula, hence $\varnothing$ $\longrightarrow$ A and B $\longrightarrow$ $\varnothing$ designate the import of A and the export of B, respectively. Note that this definition also allows catalyzed import and export reactions, e.g., C $\longrightarrow$ C + A or B + C $\longrightarrow$ C.

In thermodynamics, a system is closed if it does not exchange matter with its environment, but may exchange energy in the form of work or heat [60]. For a RN, this rules out import and export reactions.

#### **Definition 1**

A RN $(X,\mathscr {R})$ is *closed* if all reactions $r\in \mathscr {R}$ are proper.

Given an arbitrary RN $(X,\mathscr {R})$, there is a unique inclusion-maximal closed RN contained in $(X,\mathscr {R})$, namely $(X,\mathscr {R}^\text {p})$ with

2 $$\begin{aligned} \mathscr {R}^p= \{r\in \mathscr {R}\mid r \text { is proper}\}. \end{aligned}$$

We will refer to $(X,\mathscr {R}^p)$ as the *proper part* of $(X,\mathscr {R})$.

For every reaction *r*, one can define a *reverse reaction* $\overline{r}$ that is obtained from *r* by exchanging the role of products and educts. That is, $\overline{r}$ is the reverse of *r* iff, for all $x\in X$, it holds that

3 $$\begin{aligned} s^-_{x\overline{r}} = s^+_{xr} \quad \text {and}\quad s^+_{x\overline{r}} = s^-_{xr} . \end{aligned}$$

While thermodynamics dictates that every reaction is reversible in principle (albeit possibly with an extremely low reaction rate), it is a matter of modeling whether sufficiently slow reactions are included in the reaction set $\mathscr {R}$.

Chemical reactions can be composed and aggregated into “overall reactions”. In the literature on metabolic networks, *pathways* are of this form. An overall reaction consists of multiple reactions that collectively convert a set of educts into a set of products. It can be represented as a formal sum of reactions $\sum _{r\in \mathscr {R}} \mathbf {v}_r \, r$, where the vector of multiplicities $\mathbf {v}\in \mathbb {N}^\mathscr {R}_0$ has non-negative integer entries. Thereby, $[\mathbf {S}\mathbf {v}]_x$ determines the net consumption or production of compound *x* in the overall reaction specified by $\mathbf {v}$.

A vector $\mathbf {v}\in \mathbb {N}_0^\mathscr {R}$ can be interpreted as an *integer hyperflow* in the following sense: If *x* is neither an educt nor a product of the overall reaction specified by $\mathbf {v}$, then $[\mathbf {S}\mathbf {v}]_x = \sum _r (s^+_{xr}-s^-_{xr}) \mathbf {v}_r = 0$, i.e., every unit of *x* that is produced by some reaction *r* with $\mathbf {v}_r>0$ is consumed by another reaction $r'$ with $\mathbf {v}_{r'}>0$.

The effect of an overall reaction can be represented via formal sums of species in two ways: as *composite reactions*,

4 $$\begin{aligned} \sum _{x\in X} \left( \sum _{r\in \mathscr {R}} s^-_{xr}\mathbf {v}_r\right) x \longrightarrow \sum _{x\in X} \left( \sum _{r\in \mathscr {R}} s^+_{xr}\mathbf {v}_r\right) x , \end{aligned}$$

or as *net reactions*,

5 $$\begin{aligned} \begin{aligned} \sum _{x\in X}&\left( \sum _{r\in \mathscr {R}} \left[ (s^-_{xr}-s^+_{xr})\mathbf {v}_r\right] _{+}\right) x \longrightarrow \sum _{x\in X} \left( \sum _{r\in \mathscr {R}} \left[ (s^+_{xr}-s^-_{xr})\mathbf {v}_r\right] _{+}\right) x . \end{aligned} \end{aligned}$$

Here we use the notation $[c]_+ = c$ if $c>0$ and $[c]_+=0$ for $c\le 0$. In Eq. (5), intermediates, i.e., formal catalysts are cancelled. Hence, the net consumption (or production) of a species *x* is $\sum _{r\in \mathscr {R}}[(s^-_{xr}-s^+_{xr})\mathbf {v}_r]_{+}=-[\mathbf {S}\mathbf {v}]_x$ if $[\mathbf {S}\mathbf {v}]_x<0$ (or $\sum _{r\in \mathscr {R}}[(s^+_{xr}-s^-_{xr})\mathbf {v}_r]_{+}=[\mathbf {S}\mathbf {v}]_x$ if $[\mathbf {S}\mathbf {v}]_x>0$).

Fig. 1 shows the RN of Oro’s prebiotic adenine synthesis from HCN and the integer hyperflow $\mathbf {v}$ corresponding to the net reaction “5 HCN $\longrightarrow$ adenine” as an example.

While a restriction to integer hyperflows $\mathbf {v}\in \mathbb {N}_0^\mathscr {R}$ is necessary in many applications, see e.g. [21] for a detailed discussion, it appears mathematically more convenient to use the more general setting of *fluxes* $\mathbf {v}\in \mathbb {R}^\mathscr {R}_\ge$ as in the analysis of metabolic pathways. To emphasize the connection with the body of literature on network (hyper)flows we will uniformly speak of flows.

For any vector $\mathbf {v}\in \mathbb {R}^\mathscr {R}$, we write $\mathbf {v}\ge 0$ if $\mathbf {v}$ is non-negative, $\mathbf {v}>0$ if $\mathbf {v}$ is non-negative and non-zero, that is, at least one entry is positive, and $\mathbf {v}\gg 0$ if all entries of $\mathbf {v}$ are positive. Analogously, we write $\mathbf {v}\le 0$, $\mathbf {v}<0$, and $\mathbf {v}\ll 0$. In particular, a vector $\mathbf {v}\in \mathbb {R}^\mathscr {R}$ is called a *flow* if $\mathbf {v}\ge 0$.

A *non-trivial* flow satisfies $\mathbf {v}>0$, i.e., $\mathbf {v}\ne 0$. Two flows $\mathbf {v_1}$ and $\mathbf {v_2}$ are called *parallel* if they describe the same net reaction. In particular, we therefore have $\mathbf {S}\mathbf {v_1} = \mathbf {S}\mathbf {v_2}$ for parallel flows.

Futile cycles in a RN are non-trivial flows for which educts and products coincide and thus the net reaction is empty.

#### **Definition 2**

A flow $\mathbf {v}>0$ is a *futile cycle* if $\mathbf {S}\mathbf {v}=0$.

We use the term futile cycle in the strict sense to describe the concurrent activity of multiple reactions (or pathways) having no net effect other than the dissipation of energy. In the literature on metabolic networks often a less restrictive concept is used that allows certain compounds (usually co-factors, ATP/ADP, redox equivalents, or solvents) to differ between products and educts, see e.g. [61–64]. In this setting, the net reaction of concurrent glycolysis and gluconeogenesis, namely the hydrolysis of ATP, is viewed as energy dissipation rather than a chemical reaction. In our setting, ${\text{ATP}} + {{\text{H}}_{2}} {\text{O}} \longrightarrow {\text{ADP}}+ {{\text{P}}_i^{-}} + {\text{H}}^{+}$, is a net reaction like any other, and hence a futile cycle would only arise if recycling of ATP, i.e., ADP + ${\text{P}}_i^{-} + {{\text{H}}^{+}} \longrightarrow {\text{ATP}} + {{\text H}_{2}}{\text{O}}$, was included as well.

If a RN has a futile cycle, it also has an integer futile cycle $\mathbf {v}\in \mathbb {N}_0^\mathscr {R}$, since $\mathbf {S}$ has integer entries and thus its kernel has a rational basis, which can be scaled with the least common denominator to have integer entries.

A pair $(X',\mathscr {R}')$ is a *subnetwork* of $(X,\mathscr {R})$ if $X'\subseteq X$, $\mathscr {R}'\subseteq \mathscr {R}$, and ${{\,\mathrm{supp}\,}}(r)\subseteq X'$ implies $r\in \mathscr {R}'$. We say that a property *P* of a RN is *hereditary* if “$(X,\mathscr {R})$ has *P*” implies that every subnetwork “$(X',\mathscr {R}')$ has *P*”.

Chemical reactions are subject to thermodynamic constraints that are a direct consequence of the conservation of energy, the conservation of mass, and the reversibility of chemical reactions. In the context of chemistry, conservation of mass is of course a consequence of the conservation of atoms throughout a chemical reaction. In the following sections, we investigate how these physical principles constrain RNs. Since we have introduced RNs in terms of abstract molecules and reactions, Eq. (1), we express the necessary conditions in terms of the stoichiometric matrix $\mathbf {S}$, which fully captures only the proper part of the RN. Throughout this work, therefore, *we assume that* $(X,\mathscr {R})$ *is a closed RN, unless explicitly stated otherwise.*

### Thermodynamic constraints

#### Reaction energies and perpetuum mobiles

Every chemical reaction *r* is associated with a change in the Gibbs free energy of educts and products. We therefore introduce a vector of *reaction (Gibbs free) energies* $\mathbf {g}\in \mathbb {R}^\mathscr {R}$ and write $(X,\mathscr {R},\mathbf {g})$ for a RN endowed with reaction energies. The reaction energy for an overall reaction is the total energy of the individual reactions involved. In terms of $\mathbf {v}\in \mathbb {R}^\mathscr {R}$, it can be expressed as

6 $$\begin{aligned} \sum _{r\in \mathscr {R}} \mathbf {g}_r\mathbf {v}_r = \mathbf {g}^\top \mathbf {v}= \langle \mathbf {g},\mathbf {v}\rangle , \end{aligned}$$

where $\langle \cdot ,\cdot \rangle$ denotes the scalar product on $\mathbb {R}^\mathscr {R}$.

Futile cycles *may* act as a chemical version of a perpetuum mobile. This is the case whenever a flow $\mathbf {v}> 0$ with zero formal net reaction, $\mathbf {S}\mathbf {v}= 0$, increases or decreases energy, i.e., if $\langle \mathbf {g},\mathbf {v}\rangle \ne 0$.

##### **Definition 3**

Let $(X,\mathscr {R},\mathbf {g})$ be a RN with reaction energies. A flow $\mathbf {v}> 0$ is a *perpetuum mobile* if $\mathbf {S}\mathbf {v}=0$ and $\langle \mathbf {g},\mathbf {v}\rangle \ne 0$.

The classical concept of a perpetuum mobile decreases its energy, $\langle \mathbf {g},\mathbf {v}\rangle < 0$, thereby “creating” energy for its environment. An “anti” perpetuum mobile with $\langle \mathbf {g},\mathbf {v}\rangle > 0$ would “annihilate” energy. Either situation violates energy conservation and thus cannot be allowed in a chemical RN. Obviously, there is no perpetuum mobile if $(X,\mathscr {R})$ does not admit a futile cycle.

In fact, thermodynamics dictates that Gibbs free energy is a state function. Two parallel flows $\mathbf {v_1}$ and $\mathbf {v_2}$ therefore must have the same associated net reaction energies. That is, $\mathbf {S}\mathbf {v_1}=\mathbf {S}\mathbf {v_2}$ implies $\langle \mathbf {g}, \mathbf {v^1}\rangle = \langle \mathbf {g},\mathbf {v^2}\rangle$. Equivalently, any vector $\mathbf {v}=\mathbf {v^1}-\mathbf {v^2} \in \mathbb {R}^\mathscr {R}$ with $\mathbf {S}\mathbf {v}=0$ must satisfy $\langle \mathbf {g},\mathbf {v}\rangle =0$. That is, $\mathbf {g}\in (\ker \mathbf {S})^\perp$.

##### **Definition 4**

Let $(X,\mathscr {R},\mathbf {g})$ be a RN with reaction energies. Then $(X,\mathscr {R},\mathbf {g})$ is *thermodynamic* if $\mathbf {v}\in \mathbb {R}^\mathscr {R}$ and $\mathbf {S}\mathbf {v}=0$ imply $\langle \mathbf {g},\mathbf {v}\rangle =0$, that is, if $\mathbf {g}\in (\ker \mathbf {S})^\perp$.

Let $(X,\mathscr{R},\mathbf{g})$ be thermodynamic, $(X',\mathscr {R}')$ be a subnetwork of $(X,\mathscr{R})$, and $\mathbf {g}'$ be the restriction of $\mathbf {g}$ to $\mathscr {R}'$. Then $\mathbf {v}'\in \mathbb {R}^{\mathscr {R}'}$ corresponds to $\mathbf {v}\in \mathbb {R}^{\mathscr {R}}$ with ${{\,\mathrm{supp}\,}}(\mathbf {v})\subseteq \mathscr {R}'$, and thus $\mathbf {v}'\in \mathbb {R}^{\mathscr {R}'}$ and $\mathbf {S}'\mathbf {v}'=0$ imply $\mathbf {S}\mathbf {v}=0$ and further $\langle \mathbf {g}',\mathbf {v}'\rangle =\langle \mathbf {g},\mathbf {v}\rangle =0$. Hence $(X',\mathscr {R}',\mathbf {g}')$ is again thermodynamic.

We note that the reaction energies of a reaction *r* and its reverse $\overline{r}$ necessarily cancel:

##### **Lemma 5**

If *r* and $\overline{r}$ are reverse reactions in a thermodynamic network $(X,\mathscr {R},\mathbf {g})$, then $\mathbf {g}_{\overline{r}}=-\mathbf {g}_r$.

##### *Proof*

If *r* and $\overline{r}$ are reverse reactions, then $\mathbf {v}$ with $\mathbf {v}_r=\mathbf {v}_{\overline{r}}=1$ (and $\mathbf {v}_{r'}=0$ otherwise) satisfies $\mathbf {S}\mathbf {v}=0$. Thus $\langle \mathbf {g},\mathbf {v}\rangle = \mathbf {g}_r+\mathbf {g}_{\overline{r}}=0$. $\square$

#### Digression: molecular energies and Hess’ Law

Every molecular species $x\in X$ has an associated *Gibbs free energy of formation*. For notational simplicity, we write $\mathbf {G}_x$ instead of the commonly used symbol $G_\mathrm {f}(x)$. The corresponding vector of *molecular energies* is denoted by $\mathbf {G}\in \mathbb {R}^X$. Molecular energies and reactions energies $\mathbf {g}\in \mathbb {R}^\mathscr {R}$ are related by Hess’ law: For every reaction $r \in \mathscr {R}$, it holds that

$$\begin{aligned} \mathbf {g}_r = \sum _{x\in X} \mathbf {G}_x (s^+_{xr}-s^-_{xr}) = \sum _{x\in X} \mathbf {G}_x \, \mathbf {S}_{xr} . \end{aligned}$$

In matrix form, the relationship between reaction energies $\mathbf {g}$ and molecular energies $\mathbf {G}$ amounts to

7 $$\begin{aligned} \mathbf {g}= \mathbf {S}^\top \mathbf {G}. \end{aligned}$$

##### **Proposition 6**

*Let* $(X,\mathscr {R})$ *be a RN and* $\mathbf {g}\in \mathbb {R}^\mathscr {R}$ *be a vector of reaction energies. Then* $(X,\mathscr {R},\mathbf {g})$ *is thermodynamic if and only if there is a vector of molecular energies* $\mathbf {G}\in \mathbb {R}^X$ *satisfying Hess’ law, Eq*. (7).

##### *Proof*

By Definition 4, $(X,\mathscr {R},\mathbf {g})$ is thermodynamic if $\mathbf {g}\in (\ker \mathbf {S})^\perp = {{\,\mathrm{im}\,}}\mathbf {S}^\top$, that is, if there is $\mathbf {G}$ such that $\mathbf {g}= \mathbf {S}^\top \mathbf {G}$, satisfying Hess’s law. $\square$

Note that the vector of molecular energies $\mathbf {G}$ is *not* uniquely determined by $\mathbf {g}$ in general.

#### Reversible and irreversible networks

To begin with, we consider purely reversible or irreversible RNs.

##### **Definition 7**

A RN $(X,\mathscr {R})$ is *reversible* if $r\in \mathscr {R}$ implies $\overline{r}\in \mathscr {R}$ and *irreversible* if $r\in \mathscr {R}$ implies $\overline{r}\notin \mathscr {R}$.

In reversible networks, general vectors $\mathbf {v}\in \mathbb {R}^\mathscr {R}$ have corresponding flows $\mathbf {{\tilde{v}}} \ge 0$ with the same net reactions and, in the case of thermodynamic networks, with the same energies.

##### **Lemma 8**

*Let* $(X,\mathscr {R},\mathbf {g})$ *be a reversible RN (with reaction energies), and let* $\mathbf {v}\in \mathbb {R}^\mathscr {R}$ *be a vector. Then there is a flow* ${\mathbf{\tilde v}} \ge 0$ *such that* $\mathbf {S} {\mathbf {{\tilde{v}}}} = \mathbf {S}\mathbf {v}$. *If* $(X,\mathscr {R},\mathbf {g})$ *is thermodynamic, then further* $\langle \mathbf {g}, \mathbf {{\tilde{v}}} \rangle = \langle \mathbf {g}, \mathbf {v}\rangle$.

##### *Proof*

If $\mathbf {v}\ge 0$, there is nothing to show. Otherwise, there are two flows $\mathbf {v^1} \ge 0$ and $\mathbf {v^2} > 0$ such that $\mathbf {v}=\mathbf {v^1}-\mathbf {v^2}$. Since $(X,\mathscr {R})$ is reversible, each reaction $r\in \mathscr {R}$ has a reverse $\overline{r}$, and we define the reverse flow $\mathbf {{\bar{v}}^2}>0$ such that $\mathbf {{\bar{v}}^2}_{r} = \mathbf {v}^\mathbf {2}_{\overline{r}}$. By construction, it satisfies $\mathbf {S}\mathbf {{\bar{v}}^2} = - \mathbf {S}\mathbf {v^2}$.

Now consider the flow $\mathbf {{\tilde{v}}} = \mathbf {v^1}+\mathbf {{\bar{v}}^2} > 0$. It satisfies

$$\begin{aligned}\mathbf {S}\mathbf {{\tilde{v}}}= \mathbf {S}(\mathbf {v^1}+\mathbf {{\bar{v}}^2}) = \mathbf {S}(\mathbf {v^1} - \mathbf {v^2}) = \mathbf {S}\mathbf {v}.\end{aligned}$$

If the network is thermodynamic, then the reverse flow satisfies $\langle \mathbf {g}, {{\bar{\mathbf{v}}}^2} \rangle = - \langle \mathbf {g}, \mathbf {v^2} \rangle$, by Lemma 5. Hence,

$$\displaystyle \langle \mathbf {g}, \mathbf {\tilde{v}} \rangle = \langle \mathbf {g}, \mathbf {v^1} + \mathbf {{\bar{v}}^2} \rangle = \langle \mathbf {g}, \mathbf {v^1} - \mathbf {v^2} \rangle = \langle \mathbf {g}, \mathbf {v}\rangle$$. $$\square$$

By definition, a thermodynamic network cannot contain a perpetuum mobile. Conversely, by the result below, if a reversible network is not thermodynamic, then it contains a perpetuum mobile.

##### **Proposition 9**

*Let* $(X,\mathscr {R},\mathbf {g})$ *be a reversible RN with reaction energies. Then, the following two statements are equivalent:*

(i) $$(X,\mathscr {R},\mathbf {g})$$ is thermodynamic.
(ii) $(X,\mathscr {R},\mathbf {g})$ contains no perpetuum mobile.

##### *Proof*

Suppose $(X,\mathscr {R},\mathbf {g})$ is not thermodynamic. That is, there is $\mathbf {v}\in \mathbb {R}^\mathscr {R}$ with $\mathbf {S}\mathbf {v}=0$ and $\langle \mathbf {v}, \mathbf {g}\rangle \ne 0$. By Lemma 8, there is $\mathbf {{\tilde{v}}}\ge 0$ with $\mathbf {S}\mathbf {{\tilde{v}}}=0$ and $\langle \mathbf {{\tilde{v}}}, \mathbf {g}\rangle \ne 0$, that is, a perpetuum mobile. $\square$

The exclusion of a perpetuum mobile is not sufficient in non-reversible systems.

##### *Example 10*

Consider the following RN (with reaction energies $\mathbf {g}$):8It contains one futile cycle,

$${\mathrm A} {\mathop {\rightarrow }\limits ^{1}}{\mathrm B} {\mathop {\rightarrow }\limits ^{\overline{1}}}{\mathrm A}$$, $$\mathbf {v}=(1,1,0,0)^\top$$ with $$\langle \mathbf {g},\mathbf {v}\rangle = 0$$,

but no perpetuum mobile. However, it contains two parallel flows with different energies,

$${\mathrm A} {\mathop {\rightarrow }\limits ^{1}}{\mathrm B} {\mathop {\rightarrow }\limits ^{2}}{\mathrm C}$$, $$\mathbf {v}=(1,0,1,0)^\top$$ with $$\langle \mathbf {g},\mathbf {v}\rangle = -2$$,

$${\mathrm A} {\mathop {\rightarrow }\limits ^{3}}{\mathrm C}$$, $$\mathbf {v}=(0,0,0,1)^\top$$ with $$\langle \mathbf {g},\mathbf {v}\rangle = -1$$.

Hence, the RN (with reaction energies $\mathbf {g}$) is not thermodynamic. By setting $\mathbf {g}_3=-2$, it can be made thermodynamic.

Many RN models are non-reversible, i.e., they contain irreversible reactions whose reverse reactions are so slow that they are neglected. From a thermodynamic perspective, irreversible reactions *r* must be *exergonic*, i.e., $\mathbf {g}_r<0$. We first consider the extreme case that all reactions $r\in \mathscr {R}$ are irreversible.

##### **Proposition 11**

*Let* $(X,\mathscr {R},\mathbf {g})$ *be an irreversible RN with reaction energies. Then, every futile cycle is a perpetuum mobile. Hence, if* $(X,\mathscr {R},\mathbf {g})$ *is thermodynamic, then there are no futile cycles.*

##### *Proof*

Consider a futile cycle, that is, a flow $\mathbf {v}> 0$ with $\mathbf {S}\mathbf {v}=0$. Since all reactions are exergonic, $\mathbf {v}_r>0$ implies $\mathbf {g}_r<0$ and further $\langle \mathbf {g}, \mathbf {v}\rangle < 0$, that is, $\mathbf {v}$ is a perpetuum mobile. Now, if there is a futile cycle and hence a perpetuum mobile, then the network is not thermodynamic. $\square$

#### Thermodynamic soundness

We next ask whether a RN $(X,\mathscr {R})$ can always be endowed with a vector of reaction energies $\mathbf {g}$ such that $(X,\mathscr {R},\mathbf {g})$ is thermodynamic.

##### **Definition 12**

A RN $(X,\mathscr {R})$ is *thermodynamically sound* if there is a vector of reaction energies $\mathbf {g}$ such that $(X,\mathscr {R},\mathbf {g})$ is a thermodynamic network.

We note that thermodynamic soundness is a hereditary property of RNs, since we have seen above that if $(X,\mathscr {R},\mathbf {g})$ is a thermodynamic network so are all its subnetworks $(X',\mathscr {R}',\mathbf {g}')$.

Again, we first consider purely reversible or irreversible RNs.

##### **Proposition 13**

*Every reversible RN is thermodynamically sound.*

##### *Proof*

Since $\mathbf {S}\ne 0$ (the zero matrix), obviously $(\ker \mathbf {S})^\perp = {{\,\mathrm{im}\,}}\mathbf {S}^\top \ne \{0\}$ (the zero vector), and hence there is a non-zero $\mathbf {g}\in (\ker \mathbf {S})^\perp$. $\square$

##### **Theorem 14**

*An irreversible RN is thermodynamically sound if and only if there are no futile cycles.*

##### *Proof*

By Gordan’s Theorem (which is in turn a special case of Minty’s Lemma [65], see Appendix B in [66]): Either there is a negative $\mathbf {g}\in (\ker \mathbf {S})^\perp$ or there is a non-zero, non-positive $\mathbf {v}\in \ker \mathbf {S}$. That is, either there is $\mathbf {g}\ll 0$ with $\mathbf {g}\in (\ker \mathbf {S})^\perp$ (the network is thermodyn. sound) or there is $\mathbf {v}< 0$ with $\mathbf {v}\in \ker \mathbf {S}$; equivalently, there is a futile cycle $\mathbf {v}>0$. $\square$

It is not always obvious from the specification of an artificial chemistry model whether or not it is thermodynamically sound. As an example, we consider the artificial chemistry proposed in [67]. It considers only binary reactions (two educts) that produce two products, aiming to ensure conservation of particle numbers. In one variant, the networks only contains irreversible and thus exergonic reactions. It may produce, for instance, the following set of reactions:

9 $$\begin{aligned} \begin{aligned} {\mathrm{A + B}}&\longrightarrow {\mathrm{C + D}} , \\ {\mathrm{A + C}}&\longrightarrow \mathrm{{E + B}} , \\ \mathrm{{B + D}}&\longrightarrow \mathrm{{F + A}} , \\ \mathrm{{E + F}}&\longrightarrow \mathrm{{A + B}} . \end{aligned} \end{aligned}$$

Their sum corresponds to the flow $\mathbf {v}= (1,1,1,1)^\top \ge 0$ and yields the exergonic composite reaction

$$\mathrm{2A + 2B + C + D + E + F} \longrightarrow \mathrm{2A + 2B + C + D + E + F} ,$$

that is, ${\mathbf {S}}{\mathbf {v}}=0$. Thus the model admits a futile cycle composed entirely of exergonic reactions and hence a perpetuum mobile. Thus it is not thermodynamically sound.

#### Mixed networks

In many applications, RNs contain both reversible and irreversible reactions, . There are two interpretations of such models:

- In the (*lax*) sense used above, reversible reactions can be associated with arbitrary energies, while irreversible reactions are considered exergonic. That is, the reaction energies must satisfy $\mathbf {g}_{r}<0$ for $r\in \mathscr {R}_{\mathrm {irr}}$.
- In a *strict* sense, the reaction energies assigned to irreversible reactions are much more negative than the reaction energies of the reversible ones. After scaling, one requires $|\mathbf {g}_r|\le 1$ (that is, $-1 \le \mathbf {g}_r \le 1$) for $r\in \mathscr {R}_{\mathrm {rev}}$ and $|\mathbf {g}_r|\ge \gamma$ (that is, $\mathbf {g}_r \le -\gamma$) for $r\in \mathscr {R}_{\mathrm {irr}}$ and (large) $\gamma >1$. The intuition is that reactions *r* with $\mathbf {g}_r \ge \gamma$ can be neglected.

The following example shows that thermodynamic soundness differs in the lax and strict senses.

##### *Example 15*

Consider the following RN (with reaction energies $\mathbf {g}$):10for some $g>0$. It contains two futile cycles:

$${\mathrm A} {\mathop {\rightarrow }\limits ^{1}}{\mathrm B} {\mathop {\rightarrow }\limits ^{\overline{1}}}{\mathrm A}$$, $$\mathbf {v}=(1,1,0,0)^\top$$ with $$\langle \mathbf {g},\mathbf {v}\rangle = 0$$,

$${\mathrm A} {\mathop {\rightarrow }\limits ^{1}}{\mathrm B} {\mathop {\rightarrow }\limits ^{2}}{\mathrm C} {\mathop {\rightarrow }\limits ^{3}}{\mathrm A}$$, $$\mathbf {v}=(1,0,1,1)^\top$$, $$\langle \mathbf {g},\mathbf {v}\rangle = 1-2g$$.

By setting $g=1/2$, the RN can be made thermodynamic. (Then the second futile cycle is not a perpetuum mobile.)

However, the RN in (10) *cannot* be seen as the limit of a thermodynamic, reversible network $({\mathrm A}\leftrightarrow {\mathrm B}\leftrightarrow {\mathrm C}\leftrightarrow {\mathrm A})$ for large *g*. Thereby, one considers small $\mathbf {g}_1,\mathbf {g}_{\overline{1}}$ and large negative $\mathbf {g}_2,\mathbf {g}_3$ (and hence large positive $\mathbf {g}_{\overline{2}},\mathbf {g}_{\overline{3}}$, that is, negligible reverse reactions $\overline{2}, \overline{3}$). Any such (limit of a) reversible RN contains a perpetuum mobile (the second futile cycle); equivalently, it is not thermodynamic.

##### **Definition 16**

A mixed network  is *thermodynamically sound* if there are reaction energies $\mathbf {g}$ such that $(X,\mathscr {R},\mathbf {g})$ is thermodynamic and $\mathbf {g}_r<0$ for $r\in \mathscr {R}_\mathrm {irr}$.

 is *strictly* thermodynamically sound if, for all $\gamma >1$, there are reaction energies $\mathbf {g}$ such that $(X,\mathscr {R},\mathbf {g})$ is thermodynamic, $|\mathbf {g}_r| \le 1$ for $r\in \mathscr {R}_\mathrm {rev}$, and $\mathbf {g}_r<0$ with $|\mathbf {g}_r| \ge \gamma$ for $r\in \mathscr {R}_\mathrm {irr}$.

The scaling condition can also be written in the form

11 $$\begin{aligned} \min _{r\in \mathscr {R}_{\text {irr}}} |\mathbf {g}_r| \ge \gamma \max _{r\in \mathscr {R}_{\mathrm {rev}}} |\mathbf {g}_r| \quad \text {for all } \gamma >1. \end{aligned}$$

A more detailed justification for strict thermodynamic soundness in mixed networks will be given in the next subsection when considering open RNs. Here, we focus on the relationship of thermodynamic soundness and futile cycles.

##### **Theorem 17**

*A mixed RN*  *is thermodynamically sound if and only if there is no irreversible futile cycle.*

##### *Proof*

By a “sign vector version” of Minty’s Lemma: Either there is $\mathbf {g}\in (\ker \mathbf {S})^\perp$ with $\mathbf {g}_r<0$ for $r \in \mathscr {R}_\mathrm {irr}$ (the network is thermodynamically sound) or there is a non-zero $\mathbf {v}\in \ker \mathbf {S}$ with $\mathbf {v}_r \le 0$ for $r \in \mathscr {R}_\mathrm {irr}$ and $\mathbf {v}_r = 0$ for $r \in \mathscr {R}_\mathrm {rev}$; equivalently, there is a futile cycle $\mathbf {v}>0$ with ${{\,\mathrm{supp}\,}}(\mathbf {v}) \subseteq \mathscr {R}_\mathrm {irr}$. $\square$

##### **Theorem 18**

*A mixed RN*  *is strictly thermodynamically sound if and only if no futile cycle contains an irreversible reaction.*

##### *Proof*

By Minty’s Lemma: Let $\gamma >1$. Either there is $\mathbf {g}\in (\ker \mathbf {S})^\perp$ with $\mathbf {g}_r \in [-1,1]$ for $r \in \mathscr {R}_\mathrm {rev}$ and $\mathbf {g}_r \in (-\infty ,-\gamma ]$ for $r \in \mathscr {R}_\mathrm {irr}$ or there is $\mathbf {v}\in \ker \mathbf {S}$ with

12 $$\begin{aligned} \sum _{r \in \mathscr {R}_\mathrm {rev}} \mathbf {v}_r \, [-1,1] + \sum _{r \in \mathscr {R}_\mathrm {irr}} \mathbf {v}_r \, (-\infty ,-\gamma ] > 0. \end{aligned}$$

Thereby, a sum of intervals is defined in the obvious way, yielding an interval which is positive if each of its elements is positive. Via $\mathbf {v}\rightarrow -\mathbf {v}$, the interval condition (12) is equivalent to: there is $\mathbf {v}\in \ker \mathbf {S}$ with

13 $$\begin{aligned} \sum _{r \in \mathscr {R}_\mathrm {rev}} \mathbf {v}_r \, [-1,1] + \sum _{r \in \mathscr {R}_\mathrm {irr}} \mathbf {v}_r \, [\gamma ,\infty ) > 0 . \end{aligned}$$

As necessary conditions, we find (i) $\mathbf {v}_{r^*} > 0$ for some $r^* \in \mathscr {R}_\mathrm {irr}$ and (ii) $\mathbf {v}_{r} \ge 0$ for all $r \in \mathscr {R}_\mathrm {irr}$. By Lemma 8, (iii) there is an equivalent flow with $\mathbf {v}_{r} \ge 0$ for $r \in \mathscr {R}_\mathrm {rev}$. That is, there is a futile cycle $\mathbf {v}>0$ involving an irreversible reaction. For $\gamma$ large enough, the necessary conditions are also sufficient for the interval condition (13). $\square$

We may characterize strict thermodynamic soundness for mixed networks also in geometric terms:

##### *Corollary 19*

**Let** , $L_\mathrm {rev} = {{\,\mathrm{im}\,}}\mathbf {S}_\mathrm {rev}$, *and* $C_\mathrm {irr} = {{\,\mathrm{cone}\,}}\mathbf {S}_\mathrm {irr}$*. Then,* $(X, \mathscr {R})$ *is strictly thermodynamically sound if and only if it is thermodynamically sound and* $L_\mathrm {rev} \cap C_\mathrm {irr} = \{0\}$.

**Fig. 2:**
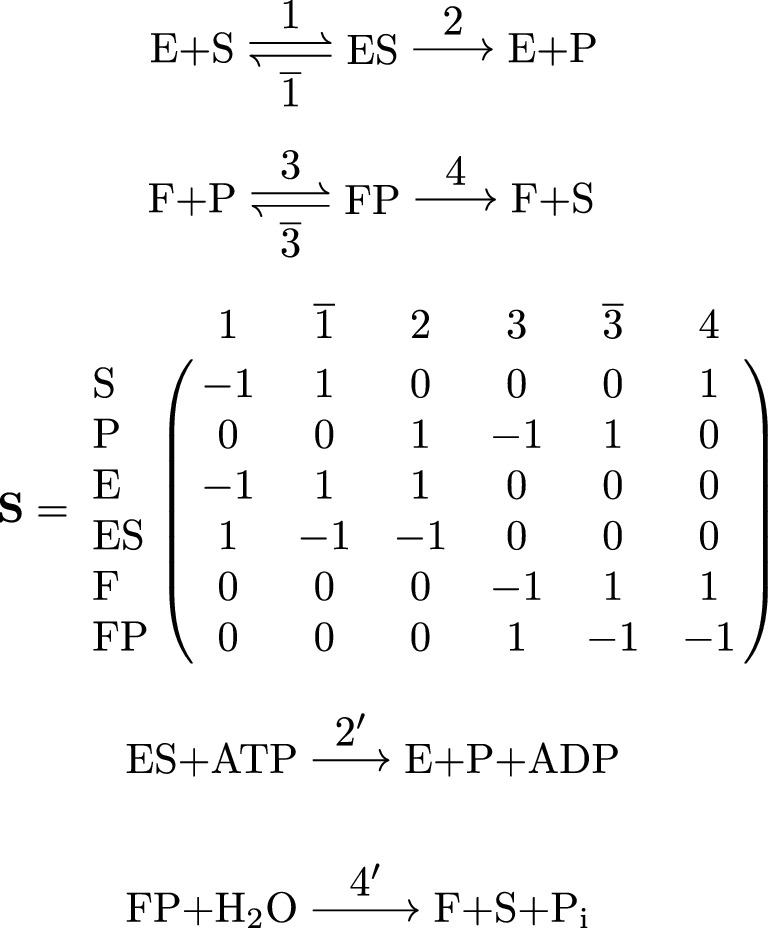
Substrate cycle. Reaction network (top) as a complex-reaction graph, involving substrate *S*, product *P*, enzymes *E*, *F*, and complexes *ES*, *FP*, and stoichiometric matrix $\mathbf {S}$ (middle). In addition to the futile cycles $(1,1,0,0,0,0)^\top$ and $(0,0,0,1,1,0)^\top$, corresponding to the two (pairs of) reversible reactions, there is a non-trivial futile cycle $\mathbf {v}= (1,0,1,1,0,1)^\top$, involving both reversible and irreversible reactions. (Note that this futile cycle is not an actual cycle of the graph.) As a result, the network is thermodynamically sound, but not strictly thermodynamically sound. In a metabolically relevant example from glycolysis/gluconeogenesis, the compounds are *S* = fructose-6-phosphate, *P* = fructose-1,6-bisphosphate, *E* = phosphofructokinase 1, and *F* = fructose-1,6-bisphosphatase, and reactions 2 and 4 involve additional compounds (bottom). As a consequence, there is no non-trivial futile cycle (in the strict sense of this work). In fact, the vector $\mathbf {v}$ above then represents the net reaction $\text{ATP} + \text{H}_{2}\text{O} \rightarrow \text{ADP} + \text{P}_\mathrm {i}$. Still, it is called a futile cycle or substrate cycle in the literature on metabolic networks. (In our approach, reactions producing/consuming the additional compounds $\text{P}_\mathrm {i}$ must be added to the network to obtain a futile cycle. Such a futile cycle involves the active reactions in $\mathbf {v}$, and hence the extended network cannot be strictly thermodynamically sound.)

Figure 2 illustrates the concepts of futile cycles and (strict) thermodynamical soundness in a metabolically relevant example.

#### Open (mixed) networks

Opening the RN, i.e., adding transport reactions alters the representation of reaction energies. We now have to consider chemical potentials involving concentrations, i.e., we replace the (Gibbs free) energies $\mathbf {G}_x$ by $\mathbf {G}_x + R\,T\ln [x]$, where [*x*] is the activity of *x*, which approximately coincides with the concentrations. A reaction *r* then proceeds in the forward direction whenenver the chemical potential of the products is smaller than the chemical potential of the educts, i.e., if

14 $$\begin{aligned} \sum _x s^+_{xr} (\mathbf {G}_x + R\,T\ln [x]) < \sum _x s^-_{xr} (\mathbf {G}_x + R\,T\ln [x])\,. \end{aligned}$$

This condition can be rewritten in terms of the reaction (Gibbs free) energies and (the logarithm of) the “reaction quotient”, see e.g. [68]:

15 $$\begin{aligned} \mathbf {g}_r < - R\,T \sum _{x\in X} s_{xr}\ln [x] \end{aligned}$$

The activities [*x*] for $x\in X$ therefore define an upper bound on the reaction energy $\mathbf {g}_r$. In an open system, (internal) concentrations may be buffered as fixed values or are implictly determined by given influxes or external concentrations [69]. Given a specification of the environment, i.e., of the transport fluxes and/or buffered concentrations, the upper bound in Eq. (15) can have an arbitrary value. Thus, if an irreversible reaction in $\mathscr {R}$ is meant to proceed forward for all conditions, it must be possible to choose $\mathbf {g}_r<0$ arbitrarily small, i.e., $|\mathbf {g}_r|$ arbitrarily large. This amounts to requiring that $(X,\mathscr {R}^p)$ is *strictly* thermodynamically sound. In many studies of reaction networks, one requires that a reaction proceeds forward in a given situation, but allows that it proceeds backward in other situations. In this (lax) interpretation of irreversibility it is sufficient to require that $(X,\mathscr {R}^p)$ is thermodynamically sound, but not necessarily strictly thermodynamically sound.

In Def. 16, we introduce (strict) thermodynamical soundness in terms of reaction energies, and in Thms. 17 and 18, we characterize it in terms of futile cycles. In a corresponding approach [70, 71], “extended” detailed balance is required for (closed) RNs with irreversible reactions at thermodynamic equilibrium. Thereby, activities [*x*], rate constants $k_+,k_-$ and equilibrium constants *K* are explicitly used to formulate Wegscheider conditions for non-reversible RNs that are limits of reversible systems. The characterization of such systems in [70] is equivalent to our results.

#### Reversible completion

As models of chemistry, non-reversible networks are abstractions that are obtained from reversible thermodynamics networks by omitting the reverse of reactions that mostly flow into one direction.

##### **Definition 20**

Let $(X,\mathscr {R},\mathbf {g})$ be a thermodynamic RN with . The reversible completion of $(X,\mathscr {R},\mathbf {g})$ is the RN $(X,\mathscr {R}^*,\mathbf {g}^*)$ with  and $\mathbf {g}^*_r=\mathbf {g}_r$ for  and $\mathbf {g}^*_{\overline{r}}= -\mathbf {g}_r$ for $r\in \mathscr {R}_\mathrm {irr}$.

##### *Lemma 21*

*If* $(X,\mathscr {R},\mathbf {g})$ *is a thermodynamic RN, then its reversible completion* $(X,\mathscr {R}^*,\mathbf {g}^*)$ *is also a thermodynamic RN.*

##### *Proof*

Let $\overline{r}\in \mathscr {R}^*$ be the reverse reaction of $r \in \mathscr {R}_\mathrm {irr}$. By Prop. 6, for every $r\in \mathscr {R}$ there is a vector $\mathbf {G}\in \mathbb {R}^X$ satisfying Hess’ law. It suffices to show that $\mathbf {G}$ still satisfies Hess’ law for $(X,\mathscr {R}^*)$. By the definition of $\overline{r}$, its reaction energy is $\mathbf {g}^*_{\overline{r}} = \sum _{x\in X} \mathbf {G}_x (s^+_{x{\overline{r}}} - s^-_{x{\overline{r}}}) = \sum _{x\in X} \mathbf {G}_x (s^-_{xr}-s^+_{xr}) = -\mathbf {g}_r$, as required by Def. 20. Thus $(X,\mathscr {R}^*,\mathbf {g}^*)$ is also thermodynamic. $\square$

The following result is an immediate consequence of Lemma 21.

##### **Proposition 22**

*If the RN* $(X,\mathscr {R})$ *is thermodynamically sound, then its reversible completion is also thermodynamically sound, and the reaction energies* $\mathbf {g}$ *can be choosen such that* $\mathbf {g}_r<0<\mathbf {g}_{\overline{r}}$ *for all* $r\in \mathscr {R}_\mathrm {irr}$.

### Mass conservation and cornucopias/abysses

Thermodynamic soundness is not sufficient to ensure chemical realism. As an example, consider the random kinetics model introduced in [72]. It assigns (a randomly chosen) energy *G*(*x*) to each $x\in X$. Each reaction *r* is defined by randomly picking a set of educts $e_r^-$ and products $e_r^+$. A possible instance of this model comprises four compounds with molecular energies $G({\mathrm A}) = -5$, $G({\mathrm B}) = -5$, $G({\mathrm C}) = -10$, and $G({\mathrm X}) = -2$, and two reactions

16 $$\begin{aligned} \mathrm{A + B} \longrightarrow \mathrm{C + X}, \quad \mathrm{C} \longrightarrow \mathrm{A + B}. \end{aligned}$$

The first reaction is exergonic with $\mathbf {g}_1=-2$ and the second has reaction energy $\mathbf {g}_2=0$. The composite reaction, obtained as their sum, is $\mathrm{A + B} \rightarrow \mathrm{A + B + X}$. Ignoring the effective catalysts A and B, the corresponding net reaction is ${\varnothing } \rightarrow \mathrm{X}$. In this universe, therefore, it is possible to spontaneosly create mass in a sequence of exergonic reactions. Reverting the signs of the energies reverts the two reactions and thus yields an exergonic reaction that makes X disappear.

We can again describe this situation in terms of flows. Recall that $[\mathbf {S}\mathbf {v}]_x$ is the net production or consumption of species *x*. The spontaneous creation or annihilation of mass thus corresponds to flows $\mathbf {v}>0$ with $\mathbf {S}\mathbf {v}>0$ or $\mathbf {S}\mathbf {v}<0$, respectively.

#### **Definition 23**

Let $(X,\mathscr {R})$ be a RN. A flow $\mathbf {v}>0$ is a *cornucopia* if $\mathbf {S}\mathbf {v}>0$ and an *abyss* if $\mathbf {S}\mathbf {v}<0$.

Systems with cornucopias or abysses cannot be considered as closed systems. The proper part of chemical reaction networks therefore must be free of cornucopias and abysses.

Since in a reversible network any vector $\mathbf {v}\in \mathbb {R}^\mathscr {R}$ can be transformed into an equivalent flow $\mathbf {{\tilde{v}}} \ge 0$ (with $\mathbf {S}\mathbf {{\tilde{v}}} = \mathbf {S}\mathbf {v}$), cf. Lemma 8, we have the following characterization.

#### **Proposition 24**

*A reversible RN is free of cornucopias and abysses if and only if there is no vector* $\mathbf {v}\in \mathbb {R}^\mathscr {R}$ *such that* $\mathbf {S}\mathbf {v}>0$.

In fact, mass conservation rules out cornucopias and abysses. More generally, a *reaction invariant* is a property that does not change over the course of a chemical reaction [8, 27, 29]. Here, we are only interested in *linear* reaction invariants, also called *conservation laws* [73], that is, quantitative properties of molecules (such as mass) whose sum is the same for educts and products.

#### **Definition 25**

A *linear reaction invariant* or *conservation law* is a non-zero vector $\mathbf {m}\in \mathbb {R}^X$ that satisfies $\sum _{x\in X} \mathbf {m}_x \, s^+_{xr} = \sum _{x\in X} \mathbf {m}_x \, s^-_{xr}$ for all reactions $r\in \mathscr {R}$, that is, $\mathbf {m}^\top \mathbf {S}=0$.

#### **Definition 26**

A RN is *conservative* if it has a positive conservation law, that is, if there is $\mathbf {m}\in \mathbb {R}^X$ such that $\mathbf {m}\gg 0$ and $\mathbf {m}^\top \mathbf {S}=0$.

By definition, a conservative network is free of cornucopias and abysses. Conversely, by the result below, if a reversible network is not conservative, then it contains a cornucopia (and an abyss).

#### **Theorem 27**

*A reversible RN* $(X,\mathscr {R})$ *is free of cornucopias and abysses if and only if it is conservative.*

#### *Proof*

By Stiemke’s Theorem (which is in turn a special case of Minty’s Lemma): Either there is a non-zero, non-negative $\mathbf {n} \in {{\,\mathrm{im}\,}}\mathbf {S}$ or there is a positive $\mathbf {m}\in ({{\,\mathrm{im}\,}}\mathbf {S})^\perp = \ker \mathbf {S}^\top$. That is, either there is $\mathbf {v}\in \mathbb {R}^\mathscr {R}$ with $\mathbf {n} = \mathbf {S}\mathbf {v}> 0$ (corresponding to a cornucopia $\mathbf {{\tilde{v}}}>0$) or there is $\mathbf {m}\gg 0$ with $\mathbf {S}^\top \mathbf {m}=0$ (as claimed). $\square$

We therefore conclude that every closed chemical RN must have a positive reaction invariant. This is no longer true if the RN is embedded in an open system and mass exchange with the environment is allowed. By construction, each transport reaction violates at least one of the conservation laws of the closed system, since $[\mathbf {m}^\top \mathbf {S}]_{r}>0$ if *r* is an import reaction and $[\mathbf {m}^\top \mathbf {S}]_{r}<0$ if it is an export reaction. As discussed e.g. in [73], opening a RN by adding import or export reactions, can only reduce the number of conservation laws and cannot introduce additional constraints. Nevertheless, a RN must be chemically meaningful when the import and export reactions are turned off. That is, its proper part $(X,\mathscr {R}^p)$ must be conservative to ensure that it has a chemical realization.

## Realizations of reaction networks

### Conservation of atoms and moieties

Molecules are composed of atoms, which are – by definition – preserved in every chemical reaction. For each atom type *a*, there is a conservation law that accounts for the number of atoms of type *a* in each compound *x*. More precisely, denote by $\mathbf {A}_{ax}\in \mathbb {N}_0$ the number of atoms of type *a* in molecule *x*, i.e., the coefficients in the *chemical sum formula* $\sum _a \mathbf {A}_{ax} \, a$ for compound *x*. (Alternatively, we may think of sum formulas as multisets of atoms.) Conservation of atom *a* in reaction *r* therefore becomes

17 $$\begin{aligned} \sum _x \mathbf {A}_{ax} \mathbf {S}_{xr} = 0 . \end{aligned}$$

For all atoms and reactions and in matrix form, this condition reads $\mathbf {A}\mathbf {S}=0$. Each row of the matrix $\mathbf {A}$ thus is a non-negative linear reaction invariant, i.e., a non-negative conservation law.

Conserved *moieties* are groups of atoms that remain intact in all reactions in which they appear [26, 28, 30]. Like atoms, they lead to non-negative integer conservation laws.

However, (the vectors representing) conserved atoms or moieties need not span the left kernel of the stoichiometric matrix $\mathbf {S}$ and need not be linearly independent. To see this, consider the following two RNs comprising a single reaction. For

18 $$\text{MgCO}_{3} \longrightarrow \text{MgO} + \text{CO}_{2}$$

with $\mathbf {S}= (-1, 1, 1)^\top$, there are only two linearly independent conservation laws, e.g. (1, 1, 0) and (1, 0, 1), corresponding to the *moieties* MgO and CO_2_, while the three vectors for the atomic composition $A_{\mathrm{Mg}}=(1,1,0)$, $A_{\mathrm{C}}=(1,0,1)$, and $A_{\mathrm{O}}=(3,1,2)$ are linearly dependent. On the other hand, as noted in [26],

19 $$\text{C}_{6}\text{H}_{5}\text{CH}_{3} + \text{H}_{2} \longrightarrow \text{C}_{6}\text{H}_{6} + \text{CH}_{4}$$

with $\mathbf {S}=(-1,-1,1,1)^\top$ has three conservation laws but only two atom types, which correspond to the conservation laws $A_{\mathrm{C}}=(7,0,6,1)$ and $A_{\mathrm{H}}=(8,2,6,4)$. E.g. the phenyl-moiety $M_{ph}=(1,0,1,0)$ or the methyl-moiety $M_{\mathrm{CH_4}}=(1,0,0,1)$ form the missing third, linearly independent conservation law. The latter example also shows that atom conservation relations are not necessarily support-minimal among the non-negative integer left-kernel vectors of $\mathbf {S}$. In fact, also (0, 1, 1, 0) and (0, 1, 0, 1) are left-kernel vectors of $\mathbf {S}$, the chemical interpretation of which is less obvious.

These examples show that key chemical properties such as atom conservation or conservation of moieties are *not* encoded in the stoichiometric matrix $\mathbf {S}$. In other words, two RNs can be isomorphic as hypergraphs but describe reactions between sets of compounds that are not isomorphic in terms of their sum formulas. For example, $\mathbf {S}=(-1,-1,1,1)^\top$ is realized by the hydroalkylation of toluene in Eq. (19), but also by the inorganic reaction

20 $$\text{ MgO} + \text{H}_{2}\text{SO}_{4} \longrightarrow \text{MgSO}_{4} + \text{H}_{2}\text{O} ,$$

having four atom conservation laws, $A_{\mathrm{Mg}}=(1,0,1,0)$, $A_{\mathrm{O}}=(1,4,4,1)$, $A_{\mathrm{H}}=(0,2,0,2)$, $A_{\mathrm{S}}=(0,1,1,0)$, and three moiety convervation laws, e.g. $M_{\mathrm{MgO}}= (1,0,1,0)$, $M_{\mathrm{H_{2}O}}= (0,1,0,1)$, and $M_{\mathrm{SO_3}}= (0,1,1,0)$.

*“Semi-positive” conservation laws* [26, 74] of a RN are the non-zero elements of the polyhedral cone

21 $$\begin{aligned} K(\mathbf {S}) = \left\{ \mathbf {y}\in \mathbb {R}^{X} \mid \mathbf {y}\mathbf {S}=0, \, \mathbf {y}\ge 0 \right\} , \end{aligned}$$

the non-negative left-kernel of $\mathbf {S}$. Thereby, $K(\mathbf {S})$ is an s-cone as defined in [75], given by a subspace (here: $\ker \mathbf {S}^\top$) and non-negativity conditions. Since the s-cone $K(\mathbf {S})$ is contained in the non-negative orthant, its extreme (non-decomposable) vectors agree with its support-minimal vectors. Further, since $\mathbf {S}$ is an integer matrix, all extreme vectors of $K(\mathbf {S})$ are positive real multiples of integer vectors.

All potential *moiety conservation laws* (MCLs) [76] for a given stoichiometric matrix $\mathbf {S}$ (but unknown atomic composition) are non-zero, integer elements of $K(\mathbf {S})$, i.e., elements of the set

22 $$\begin{aligned} {\mathcal {K}}(\mathbf {S}) = \left\{ \mathbf {y}\in \mathbb {N}_0^{X} \mid \mathbf {y}\mathbf {S}=0 \right\} \setminus \{0\} . \end{aligned}$$

Clearly, ${\mathcal {K}}(\mathbf {S})$ contains the integer extreme vectors of $K(\mathbf {S})$. Ultimately, one is interested in *minimal* MCLs, i.e., minimal elements of ${\mathcal {K}}(\mathbf {S})$, cf. [77]. (Minimal vectors are called maximal in [74].)

#### **Definition 28**

A vector $\mathbf {y}\in {\mathcal {K}}(\mathbf {S})$ is *minimal* if there is no $\mathbf {y'}\in {\mathcal {K}}(\mathbf {S})$ such that $\mathbf {y'}<\mathbf {y}$.

In fact, integer minimality and integer non-decomposability are equivalent.

#### **Proposition 29**

*Let* $\mathbf {y}\in {\mathcal {K}}(\mathbf {S})$. *The following statements are equivalent:*

1. $$\mathbf {y}$$ is minimal.
2. There are no two $\mathbf {y}', \mathbf {y}''\in {\mathcal {K}}(\mathbf {S})$ such that $\mathbf {y}=c'\mathbf {y}'+c''\mathbf {y}''$ with $c',c''\in \mathbb {N}$.

#### *Proof*

Suppose $\mathbf {y}'<\mathbf {y}$. Then $\mathbf {y}=1\cdot (\mathbf {y}-\mathbf {y}')+1\cdot \mathbf {y}'$. Conversely, suppose $\mathbf {y}=c'\mathbf {y}'+c''\mathbf {y}''$. Then $\mathbf {y}', \mathbf {y}'' < \mathbf {y}$. $\square$

Most importantly, the minimal MCLs generate all MCLs.

#### **Theorem 30**

*Every element of* ${\mathcal {K}}(\mathbf {S})$ *is a finite integer linear combination of minimal elements of* ${\mathcal {K}}(\mathbf {S})$.

#### *Proof*

By Noetherian induction on the partial order < on $\mathbb {N}^X_0$ and Proposition 29. $\square$

Knowing all minimal MCLs allows to represent the compounds *X* of a RN $(X,\mathscr {R})$ in a minimal (most coarse-grained) way.

#### **Definition 31**

The minimal moiety representation (short: *mm-representation*) of a conservative RN $(X,\mathscr {R})$ is the matrix $\mathbf {M}\in \mathbb {N}_0^{{\mathcal {M}} \times X}$, where the rows of $\mathbf {M}$ are the minimal MCLs, and ${\mathcal {M}}$ is the corresponding set of abstract moieties.

For example, consider the abstract chemical reaction

23 $$\begin{aligned} {\mathrm{A + B} \longrightarrow 2 \,\mathrm{C}} \end{aligned}$$

with $\mathbf {S}= (-1,-1,2)^\top$. There are three minimal MCLs denoted by the abstract moieties ${\mathcal {M}} = \{ \mathrm{X,Y,Z} \}$: on the one hand, $M_{\mathrm{X}}=(2,0,1)$ and $M_{\mathrm{Y}}=(0,2,1)$, which are (minimal) extreme vectors of $K(\mathbf {S})$, on the other hand, $M_{\mathrm{Z}}=(1,1,1)$, which is minimal, but not extreme. Hence, the mm-representation is given by

24 $$\begin{aligned} \mathbf {M} = \begin{pmatrix} 2&{}0&{}1\\ 0&{}2&{}1 \\ 1&{}1&{}1 \end{pmatrix}, \end{aligned}$$

and the reaction (23) can be represented as

25 $$\begin{aligned} {\mathrm{X_2Z + Y_2Z} \longrightarrow 2 \, \mathrm{XYZ}} . \end{aligned}$$

By definition, ${{\,\mathrm{im}\,}}\mathbf {M}^\top \subseteq \ker \mathbf {S}^\top$. In fact, ${{\,\mathrm{im}\,}}\mathbf {M}^\top = \ker \mathbf {S}^\top$, and hence there is an obvious lower bound for the number of minimal MCLs.

#### **Lemma 32**

*Let* $\mathbf {M}\in \mathbb {N}_0^{{\mathcal {M}} \times X}$ *be the mm-representation of a conservative RN* $(X,\mathscr {R})$ *with stoichiometric matrix* $\mathbf {S}$. *Then, *${{\,\mathrm{im}\,}}\mathbf {M}^\top = \ker \mathbf {S}^\top$ *and hence* $|{\mathcal {M}}|\ge \dim \ker \mathbf {S}^\top$.

#### *Proof*

Since the left kernel of $\mathbf {S}$ and hence $K(\mathbf {S})$ contain a positive vector, we have $\dim K(\mathbf {S}) = \dim \ker \mathbf {S}^\top {=}{:}d$. Hence, (the extreme vectors of) $K(\mathbf {S})$ and therefore also (the corresponding minimal integer vectors of) ${\mathcal {K}}(\mathbf {S})$ generate $\ker \mathbf {S}^\top$, that is, ${{\,\mathrm{im}\,}}\mathbf {M}^\top = \ker \mathbf {S}^\top$. Hence, the number of minimal MCLs is greater equal *d*, that is, $|{\mathcal {M}}|\ge \dim \ker \mathbf {S}^\top$. $\square$

By instantiating the abstract moieties $\{ \mathrm{X,Y,Z} \}$ with sum formulas (multisets of atoms), every chemical realization of the reaction can be obtained. In general, we define an instance as follows.

#### **Definition 33**

A sum formula instance (short: *sf-instance*) of a RN $(X,\mathscr {R})$ with stoichiometric matrix $\mathbf {S}$ is a matrix $\mathbf {A}\in \mathbb {N}_0^{{\mathcal {A}} \times X}$ for some non-empty, finite set ${\mathcal {A}}$ of “atoms” such that

(i) each column of $\mathbf {A}$ is non-zero, and
(ii) $$\mathbf {A} \mathbf {S}= 0$$.

Def. 33 in particular allows that $\mathbf {A}$ comprises a single row. By condition (i), this row vector is a strictly positive conservation law, which, as a linear combination of MCLs, may be chosen to be integer valued. Conversely, if $(X,\mathscr {R})$ admits an sf-instance, then the column-sum $\mathbf {m}= \mathbf {1}^\top \mathbf {A}\in \ker \mathbf {S}^\top$ is a strictly positive integer conservation law and thus in particular an sf-instance with $|{\mathcal {A}}|=1$. Taken together, we have shown the following existence result.

#### **Proposition 34**

*A RN* $(X,\mathscr {R})$ *admits an sf-instance* *if and only if it is conservative.*

The entry $\mathbf {m}_x$ of $\mathbf {m}$ can be interpreted as the total number of atoms in compound $x\in X$. In [78], a RN is called *primitive atomic* if each reaction preserves the total number of atoms. Thus a RN is primitive atomic if and only if it is conservative, cf. [78].

### Isomers and sum formula realizations

In order to gain a better understanding of sf-instances for a RN $(X,\mathscr {R})$, we consider net reactions of the form ${\mathrm X\rightarrow \mathrm Y}$ in the reversible completion of $(X,\mathscr {R})$. That is, we ask whether it is possible, in principle, to convert X into Y, irrespective of whether the conversion is thermodynamically favorable. From a chemical perspective, if such a *net isomerization reaction* exists, then X and Y must be compositional isomers. These will play a key role in our discussion of realizations of $(X,\mathscr {R})$ in terms of sf-instances.

Before we proceed, we first give a more formal account of net isomerization reactions. Recall that a net reaction derives from an overall reaction, which in turn is specified by an integer hyperflow. Instead of working explicitly in the reversible completion, we may instead consider vectors $\mathbf {v}\in \mathbb {Z}^{\mathscr {R}}$ with negative entries $\mathbf {v}_r<0$, representing the reverse of irreversible reactions $r\in \mathscr {R}$.

#### **Definition 35**

Let $(X,\mathscr {R})$ be a RN with stoichiometric matrix $\mathbf {S}$. A vector $\mathbf {v}\in \mathbb {Z}^{\mathscr {R}}$, satisfying $k{:}{=}-[\mathbf {S}\mathbf {v}]_x = [\mathbf {S}\mathbf {v}]_y\in \mathbb {N}$ for some $x,y \in X$ and $[\mathbf {S}\mathbf {v}]_z=0$ for all $z\in X\setminus \{x,y\}$, specifies a *net isomerization reaction* $k\,x\rightarrow k\,y$. Two (distinct) compounds $x,y\in X$ are *obligatory isomers* if $(X,\mathscr {R})$ admits a net isomerization reaction $k\,x\rightarrow k\,y$. We write $x\rightleftharpoons y$ if $x=y$ or *x* and *y* are obligatory isomers.

#### **Proposition 36**

*The binary relation* $x\rightleftharpoons y$ *introduced in* Def. 35*is an equivalence relation.*

#### *Proof*

By definition, $\rightleftharpoons$ is reflexive. If $\mathbf {v}$ specifies the net isomerization reaction $k\,x\rightarrow k\,y$, then $-\mathbf {v}$ specifies $k\,y\rightarrow k\,x$, and thus $\rightleftharpoons$ is symmetric. To verify transitivity, suppose $x\rightleftharpoons y$ and $y\rightleftharpoons z$, i.e., there are vectors $\mathbf {v}^1$ and $\mathbf {v}^2$ that specify the net isomerization reactions $p\,x \rightarrow p\,y$ and $q\,y \rightarrow q\,z$. Then $\mathbf {v}= q\mathbf {v}_1 + p\mathbf {v}_2$ satisfies $[\mathbf {S}\mathbf {v}]_x=-pq$, $[\mathbf {S}\mathbf {v}]_z=pq$, $[\mathbf {S}\mathbf {v}]_y=0$, and $[\mathbf {S}\mathbf {v}]_u=0$ for all $u\in X\setminus \{x,y,z\}$, and thus specifies the net isomerization reaction $(pq)\,x \rightarrow (pq)\,z$. Thus, $\rightleftharpoons$ is transitive. $\square$

The intuition is to define a *sum formula realization* of a RN as a matrix $\mathbf {A}$ that (i) is an sf-instance of the RN and (ii) assigns different atomic compositions to *x* and *y* whenever $x\not \rightleftharpoons y$, that is, whenever *x* and *y* are not isomers. In the following, we will see that such a definition both ensures chemical realism and leads to a useful mathematical description. The next result relates net isomerization reactions to the structure of $\ker \mathbf {S}^\top$ (and ultimately to compositional isomers as given by MCLs and sf-instances).

#### **Theorem 37**

*Let* $(X,\mathscr {R})$ *be a RN with stoichiometric matrix* $\mathbf {S}$*. Then* $x\rightleftharpoons y$ *if and only if* $\mathbf {m}_x=\mathbf {m}_y$ *for all* $\mathbf {m}\in \ker \mathbf {S}^\top$.

#### *Proof*

First suppose $x\rightleftharpoons y$. Then either $x=y$ (in which case the assertion is trivially true) or there is a net isomerization reaction $k\,x\rightarrow k\,y$ specified by the vector $\mathbf {v}$. Let $\mathbf {m}\in \ker \mathbf {S}^\top$. By the definition of $\mathbf {v}$, we have $0 = \mathbf {m}^\top \mathbf {S}\mathbf {v}= \sum _{z\in X} \mathbf {m}_z[\mathbf {S}\mathbf {v}]_z = \mathbf {m}_x[\mathbf {S}\mathbf {v}]_x +\mathbf {m}_y[\mathbf {S}\mathbf {v}]_y = (\mathbf {m}_x-\mathbf {m}_y)[\mathbf {S}\mathbf {v}]_x$ and $[\mathbf {S}\mathbf {v}]_x\ne 0$. Hence, $\mathbf {m}_x=\mathbf {m}_y$.

Now suppose $\mathbf {m}_x=\mathbf {m}_y$ for all $\mathbf {m}\in \ker \mathbf {S}^\top$ and consider the vector $\mathbf {w}\in \mathbb {Z}^{X}$ with $\mathbf {w}_x=-1$, $\mathbf {w}_y=1$, and $\mathbf {w}_z=0$ for all $z\in X\setminus \{x,y\}$. Clearly, $\langle \mathbf {m},\mathbf {w}\rangle =0$ for all $\mathbf {m}\in \ker \mathbf {S}^\top$, that is, $\mathbf {w}\in (\ker \mathbf {S}^\top )^\perp = {{\,\mathrm{im}\,}}\mathbf {S}$. Thus there is $\mathbf {v}\in \mathbb {R}^{\mathscr {R}}$ such that $\mathbf {w}=\mathbf {S}\mathbf {v}$. Since $\mathbf {S}\in \mathbb {Z}^{X\times \mathscr {R}}$, the solution $\mathbf {v}$ of this linear equation is rational. Writing ${{\,\mathrm{lcd}\,}}(\mathbf {v})$ for the least common denomintor of the entries in $\mathbf {v}$, we obtain the integer vector ${{\,\mathrm{lcd}\,}}(\mathbf {v})\, \mathbf {v}\in \mathbb {Z}^{\mathscr {R}}$, specifying the net isometrization reaction ${{\,\mathrm{lcd}\,}}(\mathbf {v})\, x\rightarrow {{\,\mathrm{lcd}\,}}(\mathbf {v})\, y$. By definition, $x\rightleftharpoons y$. $\square$

The proof of Thm. 37 also provides a simple algorithm to compute integer hyperflows $\mathbf {v}$ that specify net isomerization reactions and to identify the obligatory isomers: For each pair $x,y\in X$, construct $\mathbf {w}$ with $\mathbf {w}_x=-1$ and $\mathbf {w}_y=1$ being the only non-zero entries and solve the linear equation $\mathbf {S}\mathbf {v}=\mathbf {w}$. We have $x\rightleftharpoons y$ if and only if a solution exists, in which case the desired integer hyperflow is ${{\,\mathrm{lcd}\,}}(\mathbf {v})\,\mathbf {v}$.

**Fig. 3:**
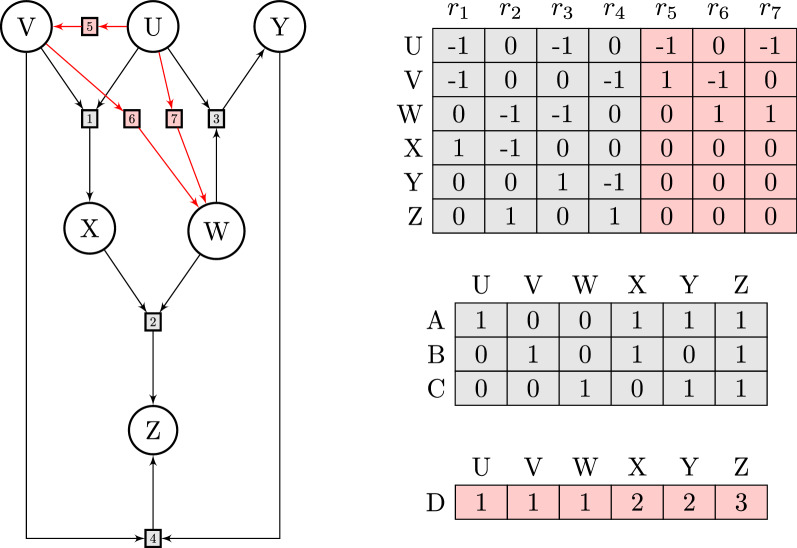
Reaction network (left) and stoichiometric matrix $\mathbf {S}$ (top right) showing reactions $r_1$-$r_4$, Eq. (26), in gray and the isomerization reactions $r_5$-$r_7$, Eq. (27) in light red. For the basic system (gray) we have $\dim \ker \mathbf {S}^\top =3$. The three MCLs are shown below $\mathbf {S}$. In the full system, $r_1$-$r_7$, we have $\dim \ker \mathbf {S}^\top =1$ with the unique MCL shown at the bottom right. In the full system U, V, and W form obligatory isomers of the monomer D. Similarly, X and Y are also obligatory isomers composed of two D units, while Z is a trimer of D units. The vector $\mathbf {v}=(-1,0,1,0,0,1,0)$ is represented by the composite reaction ${\mathrm{X + (U + W) + V} \rightarrow \mathrm{(U + V) + Y + W}}$ and specifies the net isometrization reaction ${ \mathrm X \rightarrow \mathrm Y }$

We next show that obligatory isomers cannot be distinguished by sf-instances, and conversely, compounds that are not obligatory isomers are distinguished by certain sf-instances.

#### **Theorem 38**

*Let* $(X,\mathscr {R})$ *be a RN with stoichiometric matrix* $\mathbf {S}$ *and* $\mathbf {A}\in \mathbb {N}_0^{{\mathcal {A}}\times X}$ *be an sf-instance. If* ${{\,\mathrm{im}\,}}\mathbf {A}^\top = \ker \mathbf {S}^\top$*, then the following statements are equivalent:*

(i) $x,y \in X$ are obligatory isomers;
(ii) $\mathbf {A}_{ax}=\mathbf {A}_{ay}$ for all $a\in {\mathcal {A}}$.

If ${{\,\mathrm{im}\,}}\mathbf {A}^\top \subseteq \ker \mathbf {S}^\top$, then (i) implies (ii).

#### *Proof*

Let $x,y \in X$ be distinct. On the one hand, by Theorem 37, statement (i) is equivalent to $\mathbf {m}_x=\mathbf {m}_y$ for all $\mathbf {m} \in \ker \mathbf {S}^\top$. On the other hand, statement (ii) is equivalent to $\mathbf {m}_x=\mathbf {m}_y$ for all $\mathbf {m} \in {{\,\mathrm{im}\,}}\mathbf {A}^\top$. If ${{\,\mathrm{im}\,}}\mathbf {A}^\top = \ker \mathbf {S}^\top$, then (i) and (ii) are equivalent. If ${{\,\mathrm{im}\,}}\mathbf {A}^\top \subseteq \ker \mathbf {S}^\top$, that is, if the rows of $\mathbf {A}$ are elements of $\ker \mathbf {S}^\top$, then (i) implies (ii). $\square$

Any sf-instance $\mathbf {A}$ whose rows span $\ker \mathbf {S}^\top$ not only identifies obligatory isomers, but also assigns distinct sum formulas to any distinct compounds $x,y\in X$ that are not obligatory isomers. In this case, there is at least one row (corresponding to atom *a*) for which $\mathbf {A}_{ax}\ne \mathbf {A}_{ay}$. This provides the formal justification for a mathematical definition of sum formula realizations.

#### **Definition 39**

A sum formula realization (short: *sf-realization*) of a RN $(X,\mathscr {R})$ with stoichiometric matrix $\mathbf {S}$ is a matrix $\mathbf {A}\in \mathbb {N}_0^{{\mathcal {A}} \times X}$ for some non-empty, finite set ${\mathcal {A}}$ of “atoms” such that

(i) each column of $\mathbf {A}$ is non-zero and
(ii) $${{\,\mathrm{im}\,}}\mathbf {A}^\top = \ker \mathbf {S}^\top$$.

As an illustration, consider the RN

26 $$\begin{aligned} {\mathrm{U + V} \longrightarrow \mathrm X},&\quad {\mathrm{U + W} \longrightarrow \mathrm Y}, \\ {\mathrm{X + W} \longrightarrow \mathrm Z},&\quad {\mathrm{Y + V} \longrightarrow \mathrm Z}, \end{aligned}$$

depicted on the left side of Fig. 3. The RN can be instantiated by the sum formulas ${\mathrm U} = {\mathrm A}$, ${\mathrm V}={\mathrm B}$, ${\mathrm W}={\mathrm C}$, ${\mathrm X}={\mathrm{AB}}$, ${\mathrm Y}={\mathrm{AC}}$, ${\mathrm Z} = {\mathrm{ABC}}$. The corresponding matrix $\mathbf {A}$ (middle right in Fig. 3) is not only an sf-instance, its rows also span $\ker \mathbf {S}^\top$, and hence it is an sf-realization. (In fact, it is also the mm-representation.) A “reduced representation” can be obtained by *assuming* that U, V, and W are compositional isomers corresponding to the same moiety D, that is, ${\mathrm U} = {\mathrm V} = {\mathrm W} = {\mathrm D}$. As a consequence, X and Y are also compositional isomers, ${\mathrm X} = {\mathrm Y} = {\mathrm D_2}$, and further ${\mathrm Z} = {\mathrm D_3}$. The corresponding matrix $\mathbf {A'}$ still defines an sf-instance, but its rows do not span $\ker \mathbf {S}^\top$. Now consider an extension of the RN in Eq. (26), by adding three isometrization reactions,

27 $$\begin{aligned} {{\text{U}} \longrightarrow {\text{V}}}, \quad {{\text{V}} \longrightarrow {\text{W}}}, \quad {{\text{U}} \longrightarrow {\text{W}}}. \end{aligned}$$

In the extended network given by Eq. (26) and Eq. (27), we have $\dim \ker \mathbf {S}^\top =1$, and thus there is a unique MCL. The reactions in Eq. (27) now *enforce* that U, V, and W are compositional isomers and thus correspond to the same moiety D. This coincides with the “reduced representation” $\mathbf {A'}$ for the RN in Eq. (26). The distinction is that, for the RN of Eq. (26), we may (but do not have to) assume that U, V, and W are isomers, whereas in the extended network, no other interpretation is possible.

Finally, we characterize RNs that admit an sf-realization.

#### **Proposition 40**

**A RN** $(X,\mathscr{R})$ *admits an* *sf-realization* *if and only if it is conservative.*

#### *Proof*

Suppose $(X,\mathscr {R})$ admits an sf-realization, which, in particular, is an sf-instance. By Prop. 34, $(X,\mathscr {R})$ is conservative. Conversely, suppose $(X,\mathscr {R})$ is conservative. By definition, the mm-representation is an sf-instance, and by Lemma 32, it is an sf-realization. $\square$

**Fig. 4:**
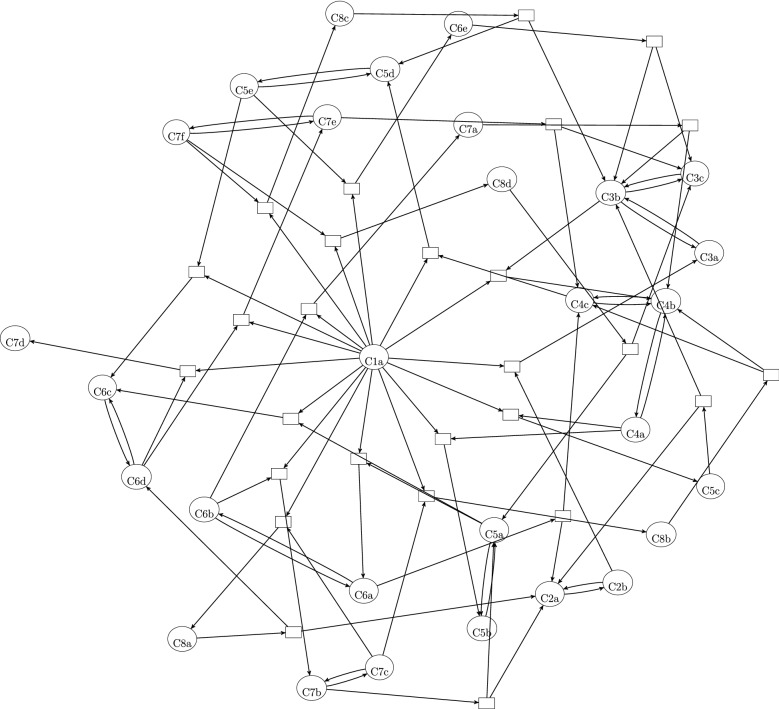
Reaction network of the formose reaction describing pre-biotic carbohydrate formation [79]. The RN is drawn here in a simplified form showing aldol and retro-aldol reactions (those with 1 educt and 2 products, and *vice versa*) without their reverse reactions. The stoichiometric matrix of the full network comprising all 38 reaction connecting the 29 compounds is provided as Addition file 1. Compounds are labeled by the number of carbon atoms. C1a (in the center) designates formaldehyd, C3c is dihydroxy acetone. The network was drawn an analyzed with MØD [21]. All compounds with the same number of carbons are obligatory isomers. Moreover, all sum formula representations are of the from $A_n$, with *A* denoting the moiety corresponding to the formaldehyd unit

Obligatory isomers put some restriction on sf-instances. Still, there is surprising freedom for sf-realizations. We say that two sf-realizations $\mathbf {A}$ and $\mathbf {A'}$ are equivalent, $\mathbf {A}\sim \mathbf {A'}$, if there are integers $p,q\in \mathbb {N}$ such that $p\mathbf {A}=q\mathbf {A'}$. One easily checks that $\sim$ is an equivalence relation. If $\dim \ker \mathbf {S}^\top =1$, then all $\mathbf {m}\in \dim \ker \mathbf {S}^\top$ are multiples of the unique minimal MCL. All sum formulas are then of the form ${D}_k$, and thus we can think of compounds simply as integers $k\in \mathbb {N}$. Every reaction thus can be written in the form $\sum _k s_{kr}^- {D}_k \rightarrow \sum _k s_{kr}^+ {D}_k$ with $\sum _k (s_{kr}^+ - s_{kr}^-)k = 0$. An example of practical interest is the rearrangement chemistry of carbohydrates, found in metabolic networks such as the pentose phosphate pathway (PPP) or the non-oxidative part of the Calvin-Benson-Bassham (CBB) cycle in the dark phase of photosynthesis. Carbohydrates may be seen as a “polymers” of formaldehyd units and can therefore be written as ${D_k =(\text{CH}_{2}\text{O})_k}$. The PPP interconverts pentoses (e.g. ribose) and hexoses (such as glucose), in an atom-economic (no waste) rearrangement network possessing the overall reaction ${6 (\text{CH}_{2}\text{O})_5 \iff 5 (\text{CH}_{2}\text{O})_6}$. In a similar fashion five 3-phosphoglycerates are reconfigured via carbohydrate chemistry into three ribulose-5-phosphate which results in the overall reaction of ${5 (\text{CH}_{2}\text{O})_3 \rightarrow 3 (\text{CH}_{2}\text{O})_5}$ if focusing on the sugar component. Carbohydrate reaction chemistry is particularly well-suited for the implementation of isomerization networks, and the logic and structure of the design space of alternative networks implementing the same overall reaction has been explored using mathematical and computational models [21, 80]. Fig. 4 shows the RN of the prebiotic carbohydrate formation according to [79]. The analysis of the corresponding stoichiometric matrix, available as Additional file 1, shows that all C*n* compounds are obligatory isomers. Furthermore, their sum formulas are necessarily multiples of the C1 unit, which corresponds to formaldehyd in the formose reaction.

For $\dim \ker \mathbf {S}^\top > 1$, there is an infinite set of sf-realizations that are pairwisely inequivalent. To see this, construct matrices $\mathbf {A}_{t} = (t_1\mathbf {y}^1,t_2\mathbf {y}^2,\ldots ,t_k\mathbf {y}^k)^\top$ from $k=\dim \ker \mathbf {S}^\top >1$ linearly independent (minimal) MCLs $\mathbf {y}^i$ and with $t\in \mathbb {N}^k$. Clearly, every such matrix $\mathbf {A}_{t}$ is an sf-realization. Furthermore, $\mathbf {A}_{t}\sim \mathbf {A}_{t'}$ if and only if there are $p,q\in \mathbb {N}$ such that $p{t}= q{t'}$. Hence $\mathbf {A}_{t}\not \sim \mathbf {A}_{t'}$ if there are two distinct indices $1\le i<j\le k$ such that $t_i / t'_i \ne t_j / t'_j$. Clearly, there is an infinite set $T \subseteq \mathbb {N}^k$ of integer vectors such that this inequality is satisfied for all distinct ${t},{t'}\in T$. For instance, one may choose distinct primes for all entries of ${t} \in T$. Thus there are infinitely many pairwisely inequivalent sf-realizations. Furthermore, the choice of the (minimal) MCLs is not unique, in general, allowing additional freedom for sf-realizations. Finally, one may produce more complex sf-realizations by appending additional rows to $\mathbf {A}$ that are linear combinations of the basis vectors. Therefore we have the following result.

#### **Proposition 41**

*Let* $(X,\mathscr {R})$ *be a conservative RN with stoichiometric matrix* $\mathbf {S}$*. If* $\dim \ker \mathbf {S}^\top >1$*, then there are infinitely many in-equivalent sf-realizations of* $(X,\mathscr {R})$.

### Structural formula realizations

**Fig. 5:**
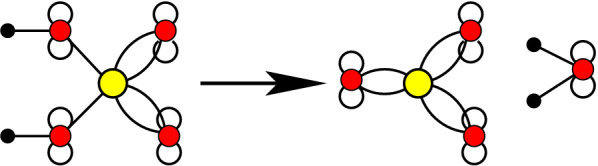
Multigraph representation for the reaction ${\text{H}_{2}\text{SO}_{4} \longrightarrow \text{SO}_{3} + \text{H}_{2}\text{O}}$. Atoms shown in color: H, black; O, red; S, yellow. Non-bonding electron pairs are represented by loops, double bonds by two parallel edges

A structural formula represents a chemical species as a (connected) molecular graph, whose vertices are labeled by atom types and edges refer to chemical bonds. *Lewis structures* [81] are equivalent to vertex-labeled multigraphs in which each bonding electron pair is represented as an individual edge, and each non-bonding electron pair as a loop. In particular, double or triple bonds are shown as two or three parallel edges. The educt and product complexes $r^-$ and $r^+$ of a reaction *r* can then be represented as the disjoint unions of the educt and product graphs, respectively. A chemical reaction is a graph transformation that converts the educt graph into the product graph such that vertices and their labels are preserved [33, 82]. Only the bonds are rearranged. Since electrons are conserved, and each edge or loop accounts for two electrons, any reaction must preserve the sum of vertex degrees and thus the number of edges. Fig. 5 shows an example.

This idea can be generalized to sf-realizations in which “atoms” are viewed as moieties. We may then interpret the vertices of a multigraph as “fragments” of species that are endowed with a certain number of “valencies” or “half bonds”. These must be “saturated” by binding to free valencies of other moieties or they must be used to form internal bonds within a moiety. In graph theory, the degree of a vertex is simply the number of incident edges. In chemistry, a related notion is the valency of an atom, i.e., the number of bonds (counting bond order) that can be formed by an atom. Each type of atom/moiety therefore has a fixed degree that we can think of as the number of halfbonds. Each of these may bind to other moieties or form a “loop”, i.e., match up with another halfbond of the same vertex. Correspondingly, the degree *d*(*u*) of a vertex *u* in a multigraph is defined as the number of edges that connect *u* with other vertices plus twice the number of loops. A reaction thus preserves electrons if and only if its only effect is to rearrange the bonds in the multigraph. The valency ${{\,\mathrm{val}}}(a)$ of an atom of type *a* is most naturally interpreted as the number of electrons in the outer shell. Loops then correspond to non-bonding electron pairs. This notion of valency matches Frankland’s “atomicity” and conforms to the IUPAC terminology [83]. Much of the chemical literature, however, uses the term valency loosely for the number of bonds; it is then not an unambigous property of an element or atom and changes with the oxidation state.

#### **Definition 42**

Let ${\mathcal {A}}$ be a non-empty, finite set, ${{\,\mathrm{val}\,}}:{\mathcal {A}}\rightarrow \mathbb {N}$ be an arbitrary function, and $\sum _{a\in {\mathcal {A}}} n_a \, a$ be a sum formula. A multigraph $\Gamma = (V,E,\alpha )$ with loops and vertex coloring $\alpha :V\rightarrow {\mathcal {A}}$ is a corresponding *structural formula* if it satisfies the following conditions:

(i) Each vertex $u\in V$ corresponds to a moiety $\alpha (u)$, in particular, $|\{u\in V:\alpha (u)=a\}|=n_a$.
(ii) $d(u)={{\,\mathrm{val}}}(\alpha (u))$ for all $u\in V$, i.e., the vertex degree of *u* is given by the corresponding moiety.
(iii) $\Gamma$ is connected.

The structural formulas specified in Def. 42 do not cover all Lewis structures. In particular, neither explicit charges nor unpaired electrons are covered. While these are important from a chemical perspective, we shall see below that such extensions are not needed for our purposes since the straightforward multigraphs in Def. 42 already provide sufficient freedom to obtain representations for all conservative RNs. Extensions to radicals and charges will be briefly considered in the Discussion section.

#### **Definition 43**

Let $(X,\mathscr {R})$ be a RN, ${\mathcal {A}}$ be a non-empty, finite set, and ${{\,\mathrm{val}}}:{\mathcal {A}}\rightarrow \mathbb {N}$ be an arbitrary function. A *Lewis instance* is an assignment of vertex-colored multigraphs $\Gamma _x=(V_x,E_x,\alpha _x)$ to all $x\in X$ such that

(i) vertex degrees satisfy $d(u)={{\,\mathrm{val}}}(\alpha _x(u))$, for all $u\in V_x$ and $x\in X$, and
(ii) the corresponding matrix $\mathbf {A} \in \mathbb {N}_0^{{\mathcal {A}}\times X}$ defined by $\mathbf {A}_{ax} = |\{ u\in V_x:\alpha _x(u)=a\}|$ is an sf-instance.

Furthermore, $x\mapsto \Gamma _x$ is a *Lewis realization* if $\mathbf {A}$ is an sf-realization.

Clearly, every Lewis realization has a corresponding sf-realization. Given an sf-realization, we therefore ask when there is a corresponding Lewis realization. By Def. 42 and 43, we have the following result.

#### **Lemma 44**

*A RN* $(X,\mathscr {R})$ *has a Lewis realization with corresponding sf-realization* $\mathbf {A}\in \mathbb {N}_0^{{\mathcal {A}}\times X}$ *for some non-empty, finite set* ${\mathcal {A}}$*, if and only if there is a function* ${\,\mathrm{val}}:{\mathcal{A}}\rightarrow \mathbb {N}$ *such that for the sum formula* $\sum _{a\in {\mathcal {A}}} \mathbf {A}_{ax} \, a$ *(for* $x\in X$*) there is a corresponding structural formula* $\Gamma _x$.

#### *Proof*

For the ’if’ part, let $\sum _{x\in {\mathcal {A}}} \mathbf {A}_{ax} \,a$ be the sum formula for $x\in X$. By assumption, there exists a vertex-colored multigraph $\Gamma _x=(V_x,E_x,\alpha _x)$ for *x* such that (i) vertex degrees satisfy $d(u)={{\,\mathrm{val}}}(\alpha _x(u))$ and (ii) the corresponding matrix equals the sf-realization $\mathbf {A}$. Analogously, for the ’only if’ part. $\square$

The appeal of this characterization is that it does not use any properties of the RN $(X,\mathscr {R})$, at all. In fact, it is easy to see that such a representation always exists.

#### **Lemma 45**

*Let* ${\mathcal {A}}$ *be a nonempty, finite set and* $\sum _{a\in {\mathcal {A}}} n_a \, a$ *be a sum formula. Then, there exists a corresponding structural formula with* ${\,\mathrm{val}}(a)=2$ *for all *$a\in {\mathcal {A}}$.

#### *Proof*

If the sum formula is given by $n_a=1$ and $n_{a'}=0$ for all $a'\in {\mathcal {A}}\setminus \{a\}$, i.e., if it is single moiety, then the corresponding structural formula is a single vertex with color *a* and a loop. Otherwise, arrange the $|V|=\sum _a n_a$ vertices, of which exactly $n_a$ are colored by *a*, in a cycle and connect the vertices along the cycle. Then every vertex *u* satisfies $d(u)={{\,\mathrm{val}}}(\alpha (u))=2$ and the graph is connected. $\square$

The result extends to any constant function ${{\,\mathrm{val}}}(a)=2k$ (with $k \in \mathbb {N}$) by adding $k-1$ loops to each vertex. As an immediate consequence of Lem. 44 and 45, we have the following result.

#### **Proposition 46**

$(X,\mathscr {R})$ *has a Lewis realization if and only if it has an sf-realization.*

Using Prop. 40, we can characterize RNs that admit a Lewis realiztion.

#### **Proposition 47**

*A RN* $(X,\mathscr {R})$ *admits a Lewis realization if and only if it is conservative*.

**Fig. 6:**
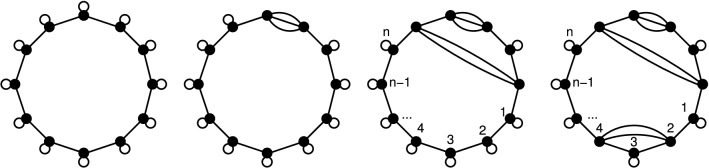
Construction of non-isomorphic multigraphs with valency 4 in the proof of Prop. 48. The first three isomers are a cycle (with loops), a cycle with a single triple-bond indicating an “origin”, and a graph with an additional double bond. In the third graph, the asymmetric arrangement of the double and triple bonds implies an unambiguous ordering of the remaining vertices (numbered from 1 to *n*). Non-isomorphic graphs are obtained converting a pair of loops into a double bound. Since each vertex has at most one bond in addition to the cycle, the resulting graphs correspond to Kleitman’s “irreducible diagrams” [84]. If crossings of bonds are excluded, the resulting induced subgraphs with vertex set $\{1,\dots ,n\}$ are isomorphic to RNA secondary structures on sequences of *n* monomers. The number $S_n$ of secondary structures grows asymptotically $\sim 2.6^n$ [85]

Interestingly, the simple multigraphs in Def. 42 are sufficient to represent all conservative RNs and thus (the proper part of) all chemical networks. Radicals and other chemical species whose structures cannot be expressed in terms of electron pairs therefore do not add to the universe of chemically realistic RNs. For more details, see the Discussion section.

Like an sf-realization, a Lewis realization does not necessarily assign distinct multigraphs $\Gamma _x$ and $\Gamma _y$ to distinct compounds *x* and *y*. In the case of sf-realizations, obligatory isomers must have the same sum formula. In Lewis realizations, however, they need not have the same multigraph.

#### **Proposition 48**

*For every conservative RN* $(X,\mathscr {R})$ *there exists *an *injective Lewis realization* $x\mapsto \Gamma _x$.

#### *Proof*

Sf-representations can be constructed to have an arbitrary number of atoms or moieties for each $x \in X$, that is, the vertex sets $V_x$ of the corresponding multigraphs $\Gamma _x$ can be chosen arbitrarily large. Set ${{\,\mathrm{val}}}(a)=4$ for all $a\in {\mathcal {A}}$ and construct an initial Lewis representation of compounds as cycles, as in the proof of Lemma 44, but with an additional loop at each vertex. Consider two obligatory isomers $x\rightleftharpoons y$, and let the (adjacent) vertices $u,v\in V_x$ be connected (by a single edge). Now replace the two loops at the corresponding vertices $u,v\in V_y$ by two additional edges between *u* and *v*. If the equivalence class of obligatory isomers contains more than two compounds, choose sets of pairs of disjoint positions along the cycles and replace pairs of loops by double edges. This yields circular matchings, familiar e.g. from the theory of RNA secondary structures [85, 86]. Setting $n=|V_x|-5$, one can construct crossing-free circular matchings on *n* vertices, whose number grows faster than $2.6^n$, see also Fig. 6. Thus, if $V_x$ is chosen large enough, an arbitrarily large set of obligatory isomers can be represented by non-isomorphic multigraphs. Note, finally, that the construction of non-isomorphic graphs does not depend on (the cardinality of) the atom set ${\mathcal {A}}$, and thus the construction is also applicable in the case $|{\mathcal {A}}|=1$, i.e., $\dim \ker \mathbf {S}^\top =1$. $\square$

The proof in particular shows that the number of vertices required to accommodate the obligatory isomers grows only logarithmically in the size of the equivalence classes of obligatory isomers.

## Discussion

### Characterization of chemistry-like reaction networks

In this contribution, we have characterized reaction networks that are chemistry-like in the sense that they are consistent with the conservation of energy and mass and allow an interpretation as transformations of chemical molecules. It is worth noting that we arrive at our results without invoking mass-action kinetics, which has been the focus of interest in chemical reaction network theory since the 1970s [7–9]. Instead, we found that basic arguments from thermodynamics (without kinetic considerations) are sufficient. The main results of this contribution can be summarized as follows:

(i) A closed RN $(X,\mathscr {R})$ is thermodynamically sound if and only if it does not contain an irreversible futile cycle. In particular, every reversible networks is thermodynamically sound. If irreversible reactions are meant to proceed in a given direction for all external conditions (after opening the RN by adding transport reactions), then $(X,\mathscr {R})$ must be *strictly* thermodynamically sound. Equivalently, a futile cycle must not contain an irreversible reaction. An analogous result was obtained by [70] assuming mass-action kinetics.
(ii) A RN $(X,\mathscr {R})$ is free of cornucopias and abysses if and only if it is conservative.
(iii) Both thermodynamic soundness and conservativity are completely determined by the stoichiometric matrix $\mathbf {S}$, i.e., they are unaffected by catalysts.
(iv) A RN $(X,\mathscr {R})$ admits an sf-realization if and only if it is conservative. That is, conservative RNs admit assignments of sum formulas such that (i) atoms (or moieties) are conserved and (ii) two compounds are assigned the same sum formula if and only if they are obligatory isomers. Obligatory isomers, in turn, are completely determined by $\mathbf {S}$.
(v) For every sf-realization of a RN $(X,\mathscr {R})$ there is also a Lewis-realization, i.e., an assignment of multigraphs to each compound such that reactions are exclusively rearrangements of edges.

Such chemistry-like realizations, however, are by no means unique. In general, the same RN has infinitely many chemical realizations corresponding to different atomic compositions. The structure of the stoichiometric matrix $\mathbf {S}$ of a closed RN therefore implies surprisingly little about the underlying chemistry.

Nevertheless there is interesting information that is independent of the concrete realization. For example, Thm. 37 can be reformulated as follows: The reversible completion of $(X,\mathscr {R})$ admits a net reaction of the form $p \, x \longrightarrow q \, y$ with $x,y\in X$ and $p,q \in \mathbb {N}$ if and only if $q \, \mathbf {m}_{x} = p \, \mathbf {m}_{y}$ for every $\mathbf {m}\in \ker \mathbf {S}^\top$. This identifies “obligatory oligomers”, necessarily composed of multiples of the same monomer.

#### Computational considerations

Somewhat surprisingly, the computational problems associated with recognizing “chemistry-like” RNs are not particularly difficult and can be solved by well-established methods. To see this, recall that $(X,\mathscr {R})$ is conservative iff there is a vector $\mathbf{m }\gg 0$ such that $\mathbf {S}^\top \mathbf{m }=0$ and *not* thermodynamically sound iff there is a vector $\mathbf {v}> 0$ such that $\mathbf {S}\mathbf {v}=0$ and $\mathbf {v}_r> 0$ for some $r\in \mathscr {R}_{\mathrm {irr}}$ These linear programming problems can be solved in $O((|X|+|\mathscr {R}|)^{2.37})$ time [87].

An integer (not necessarily non-negative) basis of $\ker \mathbf {S}^\top$ can be computed exactly in polynomial time, e.g. using the Smith normal form, see [88]. Chubanov’s algorithm finds exact rational solutions to systems of linear equations with a strict positivity constraint. Thus is can be employed to compute a strictly positive integer solution $\mathbf {m}\gg 0$ to $\mathbf {S}^\top \mathbf {m}=0$ in polynomial time [89, 90]. As a consequence, an sf-realization can also be computed explicitly in polynomial time. Each sum formula in turn can be converted into a graph with total effort bounded by $\max _{x\in X} \sum _{a}\mathbf {A}_{xa}\cdot |X|$, the maximal number of atoms that appear in a sum formula times the number of molecules.

The equivalence relation $\rightleftharpoons$ for obligatory isomers is determined by the existence of solutions to a linear equation of the form $\mathbf {S}\mathbf {v}=\mathbf {w}$ and thus can also be computed in polynomial time, again bounded by the effort for matrix multiplication for each pair $x,y \in X$. A much more efficient approach, however, is to compute a basis of $\ker \mathbf {S}^\top$, from which $\rightleftharpoons$ can be read off directly. This approach easily extends to “obligatory oligomers.”

Treating RNs as closed systems is too restrictive to describe metabolic networks. There, RNs are considered as open systems that allow the inflow of nutrients and the outflow of waste products. Models of metabolism often impose a condition of *viability*. Traditionally, this is modeled as a single export “reaction” $r_{bm}$ of the form $\sum _i \alpha _i {C}_i \rightarrow \varnothing$, known as the *biomass function* [91]. It comprises all relevant precursor metabolites ${C}_i$ (forming all relevant macromolecules) in their empirically determined proportions $\alpha _i$. Viability is then defined as the existence of a flow $\mathbf {v}>0$ with $\mathbf {S}\mathbf {v}=0$ and $\mathbf {v}_{bm}>0$. This linear programming problem can be tested efficiently by means of flux balance analysis (FBA) [92]. In contrast to $(X,\mathscr {R})$ being conservative and thermodynamically sound, however, viability is a property of the metabolic model, not of the underlying representation of the chemistry.

### Outlook to open problems

#### Construction of random chemistry-like networks

The formal characterization of chemistry-like RNs developed here suggests several interesting questions for further research. In particular, our results define rather clearly how *random* chemistry-like RNs should be defined and thus poses the question whether there are efficient algorithms for their construction. Let us consider the task of generating a random chemistry-like RN in a bit more detail. We first note that it suffices to generate a stoichiometric matrix $\mathbf {S}\in \mathbb {N}_0^{X\times \mathscr {R}}$ that is thermodynamically sound and conservative. If explicit catalysts are desired, they can be added to a reaction without further restrictions. More precisely, given $\mathbf {S}$, we obtain a network with the same stoichiometric matrix *plus* catalysts by setting

28 $$\begin{aligned} \begin{aligned} s_{xr}^- = c_{xr}, \; s_{xr}^+ = c_{xr}+s_{xr}&\quad \text {if } s_{xr}\ge 0 , \\ s_{xr}^- = c_{xr}-s_{xr}, \;s_{xr}^+ = c_{xr}&\quad \text {if } s_{xr}\le 0 . \end{aligned} \end{aligned}$$

The “catalyst matrix” $\mathbf {C}$ may contain arbritrary integers $c_{xr}\ge 0$. For the generation of a RN $(X,\mathscr {R})$, therefore, it can be drawn independently of $\mathbf {S}$.

The key task of generating $(X,\mathscr {R})$ is therefore the construction of an $|X|\times |\mathscr {R}|$ integer matrix $\mathbf {S}$ that is conservative and thermodynamically sound. Both conditions amount to the (non)existence of vectors with certain sign patterns in $\ker \mathbf {S}$ and $\ker \mathbf {S}^\top$, respectively. In order to obtain a background model for a given chemical RN, one might also ask for a random integer matrix that has a given left nullspace and is thermodynamically sound. In addition, one would probably like to (approximately) preserve the fraction of zero entries per row and column and the mean of the non-zero entries. To our knowledge, no efficient exact algorithms for this problem are known.

A potentially promising alternative is the independent generation of the complex matrix $\mathbf {Y}$ and the incidence matrix $\mathbf {Z}$ of the complex-reaction graph. Given a fixed conservative and thermodynamically sound RN, furthermore, one can make use of the heredity of thermodynamic soundness and conservativity and consider random subnetworks. This approach has been explored in particular for metabolic networks: The ensemble of viable metabolic networks in a given chemical RN can then be sampled by a random walk on the set of reactions [57] or a more sophisticated Markov-Chain-Monte-Carlo procedure [55, 93].

#### Chemistry-like realizations

The structural formulas constructed in Lemma 45 are not very “realistic’ from a chemical perspective. It is of interest, therefore, if one can construct chemically more appealing (multi-)graphs. As noted in the Introduction, the problem of designing a “molecular implementation” of a prescribed stoichiometric matrix $\mathbf {S}$ is a key problem in utilizing chemical reaction networks as computing devices. From a mathematical point of view there seem to be only a few constraints: (i) If a moiety *a* appears in isolation, i.e., as a molecule $x=1a$, then ${{\,\mathrm{val}}}(a)$ must be even, since it contains ${{\,\mathrm{val}}}(a)/2$ loops. (ii) The case ${{\,\mathrm{val}}}(a)=1$ is only possible if there is no compound composed exclusively of three or more copies of *a* or composed of more than two moieties with valency 1. (iii) It is well known that the sum of degrees must be even for every multigraph, and connectedness implies $\sum _u {{\,\mathrm{val}}}(u)\ge 2(|V|-1)$ [94].

The problem of finding multigraph realizations is closely related to, but not the same as, the problem determining the realizability of degree sequences in graphs [95] or multigraphs [96]. As in graph theory, it seems to be of particular interest to study realizability by structural formula in the presence of additional constraints on admissible graphs. Complementary to constraints on the multigraphs that render them plausible chemical graphs, the “chemical implementation” of a given $\mathbf {S}$ also involves constraints on the admissible (types of) reactions, i.e., the allowed rearrangements of edges in the multigraphs. It is much less clear how to formalize this aspect, although there seems to be a connection to graph grammar models of chemical reactions [97].

**Fig. 7:**
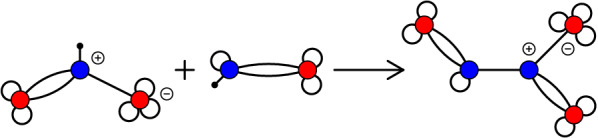
A Lewis structure-like presentation of ${\text{NO}_{2} + \text{NO} \longrightarrow \text{N}_{2}\text{O}_{3}}$ highlights that multigraphs with atom-type dependent degrees are not sufficient to represent all molecules of interest. To represent NO_2_, both an unpaired electron (shown as a semi-edge ending in a small black ball), an *N* atom with vertex degree $4<{{\,\mathrm{val}}}({N})=5$ and an oxygen atom with vertex degree $7>{{\,\mathrm{val}}}({O})=6$ are required. Similarly, *NO* is a neutral stable radical, with an unpaired electron at *N*. The product *N*2*O*3 has no unpaired electrons, but exhibits an *O* and an *N* atom with a deviant vertex degree and thus a net charge. Differences between nominal valency and actualy vertex degree are indicated by the charge symbols $\oplus$ and $\ominus$. In general, the net charge at a vertex *v* is given by ${{\,\mathrm{val}}}(\alpha (v))-\deg (v)$

An advantage of considering the multigraphs specified in Def. 42 instead of the full range of Lewis structures is that a well-established mathematical theory is available. However, “multigraphs with semi-edges”, which are essentially equivalent to Lewis structures of radicals, have been studied occasionally in recent years [98, 99] and may be an appealing framework, in particular, when restricted realizations are considered. The example of nitrogen oxids in Fig. 7 shows, however, that unpaired electrons (as in the Lewis structure of *NO*) are not the only issue. A complete implementation of Lewis structures also requires local net charges ${{\,\mathrm{val}}}(\alpha (v))-\deg (v)$ at vertices *v*, as a semi-edge-like annotation distinct from unpaired electrons, see e.g. [100].

#### Infinite RNs

Throughout this contribution, we have assumed that $(X,\mathscr {R})$ is finite. In general, however, chemical universes are infinite, at least in principle. The simplest example of infinite families are polymers. It is of interest, therefore, to develop a theory of infinite reaction networks. To this end, one could follow e.g. [101], where also infinite directed hypergraphs are considered, and further extend the literature on countably infinite undirected hypergraphs, see e.g. [102, 103] and the references therein. Most previous work pre-supposed *k*-uniformity, i.e., hyper-edges of (small) finite cardinality, matching well with the situation in chemical RNs. Every sub-RN of an infinite RN induced by a finite vertex set $Y\subset X$ can be assumed to support only a finite number of reactions (directed hyperedges) $\mathscr {R}_Y\subset \mathscr {R}$. This amounts to assuming that a sub-RN induced by finite set of compounds *Y* is a finite RN. Every finite sub-RN of a “chemistry-like” infinite RN, furthermore, needs to be conservative and thermodynamically sound. Infinite RNs will not be locally finite, in general, since every compound $x\in X$ may have infinitely many reaction partners, e.g., all members of a polymer family. Thus *x* may appear in an infinite number of reactions. These simple observations suggest infinite “chemistry-like” RNs are non-trivial structures whose study may turn out to be a worth-while mathematical endeavor.

### Supplementary Information

**Additional file 1.** Stoichiometric matrix of the complete formose RN, Fig. 4, in machine-readable form.

## Data Availability

The stoichiometric matrix of the complete formose RN, Fig. 4, is availble as machine-readable Additional file 1.

## Appendix: Mathematical notation

We consider matrices and vectors indexed by chemical species $x\in X$ or chemical reactions $r\in \mathscr {R}$. Hence, both species and reactions can be thought of as endowed with an arbitrary, but fixed order determining the order of rows and columns. Standard mathematical notation is used without further explanation in the main text. Notation that may be less familiar is summarized here:

**Table:**

$\mathbb {N}$	positive integers
$\mathbb {R}$	real numbers
$\mathbf {A}^{\!\top }$	transpose of matrix ${\mathbf {A}}$
$\ker \mathbf {A}$	kernel of matrix ${\mathbf {A}}$,
	i.e., $\ker \mathbf {A} = \{\mathbf {x} \mid \mathbf {A} \mathbf {x}= 0\}$
${{\,\mathrm{im}\,}}\mathbf {A}$	image of matrix ${\mathbf {A}}$,
	i.e., ${{\,\mathrm{im}\,}}\mathbf {A} = \{\mathbf {y} \mid \mathbf {y} = \mathbf {A}\mathbf {x} \text { for some } \mathbf {x}\}$
${{\,\mathrm{cone}\,}}\mathbf {A}$	polyhedral cone induced by matrix ${\mathbf {A}}$,
	i.e., ${{\,\mathrm{cone}\,}}\mathbf {A} = \{\mathbf {y} \mid \mathbf {y} = \mathbf {A}\mathbf {x} \text { for some } \mathbf {x}\ge 0\}$
$\mathbf {x}^{\!\top }$	row vector of column vector $\mathbf {x}$
$\mathbf {x}_i$	component of vector $\mathbf {x} \in \mathbb {R}^I$ (with $i \in I$)
${{\,\mathrm{supp}\,}}\mathbf {x}$	support of vector $\mathbf {x} \in \mathbb {R}^I$,
	i.e., ${{\,\mathrm{supp}\,}}\mathbf {x} = \{ i \in I \mid \mathbf {x}_i \ne 0 \}$
$\dim V$	dimension of vector space *V*
$V^\perp$	orthogonal complement of vector space *V*,
	i.e., $V^\perp = \{\mathbf {y} \mid \mathbf {x}^{\!\top }\mathbf {y}=0 \text { for all } \mathbf {x}\in V\}$
